# Delineating the Cytogenomic and Epigenomic Landscapes of Glioma Stem Cell Lines

**DOI:** 10.1371/journal.pone.0057462

**Published:** 2013-02-28

**Authors:** Simona Baronchelli, Angela Bentivegna, Serena Redaelli, Gabriele Riva, Valentina Butta, Laura Paoletta, Giuseppe Isimbaldi, Monica Miozzo, Silvia Tabano, Antonio Daga, Daniela Marubbi, Monica Cattaneo, Ida Biunno, Leda Dalprà

**Affiliations:** 1 Department of Surgery and Translational Medicine, University of Milan-Bicocca, Monza, Italy; 2 Science and Technology Park, Istituti di Ricovero e Cura a Carattere Scientifico (IRCCS) MultiMedica, Milan, Italy; 3 Department of Surgical Pathology, S. Gerardo Hospital, Monza, Italy; 4 Department of Pathophysiology and Organ Transplant, University of Milan, Milan, Italy; 5 Pathology Unit, Fondazione IRCCS Ca' Granda, Ospedale Maggiore Policlinico, Milan, Italy; 6 Department of Hematology-Oncology, Istituti di Ricovero e Cura a Carattere Scientifico (IRCCS) Azienda Ospedaliera Universitaria San Martino- Istituto Scientifico Tumori (IST) Istituto Nazionale per la Ricerca sul Cancro, Genova, Italy; 7 Department of Experimental Medicine, University of Genova, Genova, Italy; 8 Institute of Genetics and Biomedical Research-National Research Council, Milan, Italy; NIH/NCI, United States of America

## Abstract

Glioblastoma multiforme (GBM), the most common and malignant type of glioma, is characterized by a poor prognosis and the lack of an effective treatment, which are due to a small sub-population of cells with stem-like properties, termed glioma stem cells (GSCs). The term “multiforme” describes the histological features of this tumor, that is, the cellular and morphological heterogeneity. At the molecular level multiple layers of alterations may reflect this heterogeneity providing together the driving force for tumor initiation and development. In order to decipher the common “signature” of the ancestral GSC population, we examined six already characterized GSC lines evaluating their cytogenomic and epigenomic profiles through a multilevel approach (conventional cytogenetic, FISH, aCGH, MeDIP-Chip and functional bioinformatic analysis). We found several canonical cytogenetic alterations associated with GBM and a common minimal deleted region (MDR) at 1p36.31, including CAMTA1 gene, a putative tumor suppressor gene, specific for the GSC population. Therefore, on one hand our data confirm a role of driver mutations for copy number alterations (CNAs) included in the GBM genomic-signature (gain of chromosome 7- EGFR gene, loss of chromosome 13- RB1 gene, loss of chromosome 10-PTEN gene); on the other, it is not obvious that the new identified CNAs are passenger mutations, as they may be necessary for tumor progression specific for the individual patient. Through our approach, we were able to demonstrate that not only individual genes into a pathway can be perturbed through multiple mechanisms and at different levels, but also that different combinations of perturbed genes can incapacitate functional modules within a cellular networks. Therefore, beyond the differences that can create apparent heterogeneity of alterations among GSC lines, there’s a sort of selective force acting on them in order to converge towards the impairment of cell development and differentiation processes. This new overview could have a huge importance in therapy.

## Introduction

Glioblastoma multiforme (GBM) is the most common and lethal type of malignant brain tumor, defined as grade IV astrocytoma (WHO classification) [1]. Despite aggressive multimodal therapies, such as surgical resection, chemo- and radio-therapy, the median survival of patients is currently 15 months, according to recently reported data [2], [3], because of rapid tumor recurrence 4,5. The term “multiforme” describes the histological features of this tumor, i.e. the presence of cellular and morphological heterogeneity and the parallel coexistence of cell populations with different grades of differentiation [6]. The search for the origin of this heterogeneity, that characterizes many tumors as well as GBM, has drawn a lot of interest, also for the important implications it may have in the therapeutic field. Several cellular mechanisms have been postulated: i) in the clonal evolution model, stochastic genetic or epigenetic changes confer a selective growth advantage [7], so tumor cells in a dominant clone possess similar tumorigenic potential; ii) the cancer stem cell (CSC) model conversely claims a hierarchical organization of cells, where only a small subset of cells are tumorigenic and generate heterogeneity through differentiation [6]. These cells are endowed with stem-like properties and have been isolated from many types of tumors, including GBM, where they are termed glioma stem cells (GSCs) [8]–[12]. Although this model first seemed to be the most reliable, because it provided an explanation for resistance to both radiation and chemotherapy and eventual tumor relapse [13], [14], recent observations highlighted many complexities and uncertainties that undoubtedly deserve attention (see the recent reviews [15]–[17]). Several issues discussed include the robustness of CSC markers (which can lead to underestimate the frequency of tumorigenic cells), the variability of the CSC phenotype between patients and the presence within a tumor of multiple phenotypically or genetically distinct CSCs that coexist in a dynamic state, as tumorigenic and non-tumorigenic states can reversibly interconvert. Ultimately an emerging consensus in the field assumes that the CSC and the clonal evolution models can be interacting sources of heterogeneity [17]–[19]. Furthermore, in order to define a CSC, the cellular state and the molecular signature are much more important than the phenotype [15].

Indeed, at the molecular level multiple layers of alterations may reflect this heterogeneity: DNA mutations, chromosomal aberrations, loss of heterozygosity (LOH), copy number alterations (CNAs) and DNA methylation changes provide together the driving force for tumor initiation and development [20]. Consequently, every single level should be integrated in order to obtain a comprehensive knowledge on the multiple grades of aberrations peculiar for GBM [21]. Many GBM-related genomic alterations have been identified in the past 20 years [20], [22]–[24], but investigations that focus on the stem-like counterpart are only a few [25]–[30]. Overwhelming evidences prove that GSC lines represent the proper biological cancer model of GBM, as they are more representative of the respective primary tumor [27], [28], [30], [31].

Therefore, we deeply examined six already characterized GSC lines from the genetic and epigenetic point of view, investigating chromosomal abnormalities, LOH, CNAs and DNA methylation profiles, searching for a common “signature” specific for the ancestral GSC population. Indeed, the identification of cytogenomic and epigenomic landscapes of GSC lines is instrumental to delineate increasingly robust molecular signatures of these dynamic and complex subpopulations. The results from this and other similar studies will help to better define new potential strategies targeting GSC molecular pathways to overcome their resistance to radio- and chemo-therapy, block their function or induce their differentiation.

## Materials and Methods

### Cell Lines, Cell Culture Conditions and Patient Samples

All the cell lines used in this study have already been published. Glioma stem cell (GSC) lines were isolated from glioblastoma except one, the G179, which derives from a giant cell glioblastoma. GBM2 and GBM7 cell lines were kindly provided by the National Institute for Cancer Research, Department of Hematology-Oncology, Genova (Italy), while G144, G166, G179 and GliNS2 cell lines were kindly provided by Professor A. Smith of the Wellcome Trust – Medical Research Council Stem Cell Institute, University of Cambridge, Cambridge (UK). All the GSC lines have been extensively characterized for their stem cell properties (sphere forming assays, evaluation of differentiation properties, marker expression, *in vivo* engraftment) [32], [33]. Also the two human foetal neural stem cell lines, CB660 and CB660SP, derived from the forebrain and the spinal cord respectively, were kindly provided by Professor A. Smith [34]. These two cell lines showed a normal female karyotype 46,XX. Cell expansion was carried out in a proliferation permissive medium composed by DMEM F-12 and Neurobasal 1∶1, B-27 supplement without vitamin A (Life Technologies Italia, Milan, Italy), 2 mM L-glutamine (Euroclone S.p.A., Milan, Italy), 10 ng/ml recombinant human bFGF and 20 ng/ml recombinant human EGF (Miltenyi Biotec, Bergish Gladbach, Germany), 20 UI/ml penicillin and 20 µg/ml streptomycin (Euroclone S.p.A., Milan, Italy). GSCs and human foetal NSCs were cultured in adherent culture condition in T-25 cm^2^ flasks coated with 10 µg/ml laminin (Life Technologies Italia, Milan, Italy), in 5% CO_2_/95% O_2_ atmosphere.

Formalin-fixed paraffin embedded (FFPE) tissues of GBM tumors were derived from five-post-mortem GBM specimens and provided by the Department of Surgical Pathology, S. Gerardo Hospital, Monza (Italy).

### Immunofluorescence

The immunofluorescence assays were performed on all GSC lines using rabbit anti-CD133 (Santa Cruz Biotechnology, Santa Cruz, CA, USA; 1∶50) and mouse anti-nestin (Millipore, Billerica, MA, USA; 1∶50) as primary antibodies. Cells were placed onto slides by means of Cytospin, washed with Dulbecco’s modified phosphate-buffered saline (PBS), fixed with 4% paraformaldehyde for 15 minutes and treated for 10 minutes with 0.1 M glycine (in PBS). Slides were incubated 30 minutes at room temperature (RT) in blocking solution (5% Bovine serum albumin, BSA, 0.6% Triton X-100 in PBS) and treated for 30 minutes with 70 U/mg RNAse (Sigma Aldrich, Milan, Italy; 1∶30) in blocking solution. Cells were incubated with the primary antibodies at 4°C overnight. Then, slides were rinsed with washing buffer (0.3% Triton X-100 in PBS) and incubated with secondary fluorescent antibodies and 2.5 mg/ml propidium iodide (PI) for 1h at RT. Alexa Fluor 488-conjugated goat anti-mouse or anti-rabbit (Molecular Probes Eugene, OR, USA; 1∶200) were used as secondary antibodies. Alexa Fluor 647-conjugated phalloidin (Molecular Probes, Eugene, OR, USA; 1∶200) was used to visualize the actin filaments. Then, cells were washed with PBS and coverslips were mounted using Polyvinyl alcohol mounting medium (Fluka Analytical, Milan, Italy). Fluorescent cell preparations were examined using a Radiance 2100 confocal microscope (Bio-Rad, Hercules, CA, USA), evaluating 100 cells for each sample. Noise reduction was achieved by Kalman filtering during acquisition.

### Conventional Cytogenetics

Metaphase chromosome spreads were obtained using standard procedures. Briefly, cell cultures were treated with 0.2 µg/ml Colcemid (Roche, Basel, Switzerland) and then harvested and incubated with a hypotonic solution of 0.56% w/v KCl for 15 minutes at RT. Then, cells were fixed with fixative solution composed of 3∶1 methanol:acetic acid. The chromosomes were QFQ-banded using quinacrine mustard (Roche, Basel, Switzerland) and slides were mounted in McIlvaine buffer. Slides were analyzed using Nikon Eclipse 80i fluorescence microscope (Nikon, Amstelveen, The Netherlands) equipped with a COHU High Performance CCD camera. The number of metaphases analyzed depended on the quality of chromosome preparations. The karyotype was defined according to the guidelines of the International System for Chromosome Nomenclature 2009 (ISCN 2009). Therefore, only the clonal chromosomal abnormalities were reported. Structural rearrangement and chromosome gain must be found in at least two metaphases, whereas chromosome loss must be present in at least three cells, in order to be considered clonal. A minimum of 12 and a maximum of 34 metaphases were evaluated for each GSC line and the analysis was performed on at least 3 different passages.

### Fluorescence in situ Hybridization (FISH)

FISH was performed on metaphase chromosome spreads using whole chromosome painting (wcp) probes. Specifically, Octochrome Chromoprobe Multiprobe System (Cytocell, Cambridge, UK) was used and the procedures were assessed according to the manufacturer’s protocol. A minimum of 10 metaphases were evaluated for each specific square.

### DNA Extraction from Cell Lines and FFPE Tissues

DNA extraction from cell lines was performed using the Wizard Genomic DNA Purification Kit (Promega, Milan, Italy), according to the manufacturer’s protocol. GBM FFPE tissues were processed for DNA isolation and purification with ReliaPrep™ FFPE gDNA Miniprep System (Promega, Milan, Italy).

### Array Comparative Genomic Hybridization (aCGH)

Sample preparation, slide hybridization and analysis were performed using Human Genome CGH Microarray, 4x44K (Agilent Technologies, Santa Clara, CA, USA), according to the manufacturer’s instructions. Sex-matched commercial DNA samples (Promega, Milan, Italy) were used as reference DNA during array-CGH. Data were analyzed as previously described [35]. Briefly, the arrays were scanned at 2 µm resolution using Agilent microarray scanner and analyzed using Feature Extraction v10.7 and Agilent Genomic Workbench v5.0 software (Agilent Technologies, Santa Clara, CA, USA). The Aberration Detection Method 2 (ADM2) algorithm was used to compute and assist the identification of aberrations for a given sample (threshold = 5; log2 ratio = 0.3). The estimated percentage of mosaicism was calculated using the formula determined by Cheung SW et al. [36].

### Microsatellite Analysis

Loss of heterozygosity (LOH) analysis of chromosome 1p36-p35, chromosomes 10 and 13 was assessed by means of PCR-based assays. Amplimers were selected on the basis of their heterozygosity rate in the population and they are listed in Table S1. Amplification of each microsatellite was done in 20 µl volume with 20 ng/ml of genomic DNA, 1X PCR Buffer, 1 µM primers, 200 µM dNTPs, 1.5 mM MgCl_2_ and 1 unit of AmpliTaq Gold DNA Polymerase (Applied Biosystems, Carlsbad, CA, USA). Amplification products were resolved on 6% polyacrylamide gels and electrophoresed for 5hs at 160V. Gels were stained with 0.1% ethidium bromide and LOH was determined by visual observation.

### MeDIP-Chip

Methylated DNA immunoprecipitation and chip hybridization were performed following the guidelines of Agilent Microarray Analysis of Methylated DNA Immunoprecipitation Protocol (Version 1.0, Agilent Technologies, Santa Clara, CA, USA). Briefly, purified genomic DNA was sonicated to fragments of 200–600 bp in size and 5 µg of sheared DNA was immunoprecipitated using 50 µl of pan-mouse IgG Dynal magnetic beads (Life Technologies Italia, Monza, Italy) and 5 µg of 5-methylcytosine antibody (Eurogenetec, Seraing, Belgium). DNA was eluted and then purified by phenol: chloroform procedure and precipitated with ethanol. Neither MeDIPed DNA nor reference DNA were amplified but they were directly labeled with Cyanine 5- and Cyanine 3-dUTP nucleotides, respectively, using Agilent Genomic DNA labeling Kit Plus (Agilent Technologies, Santa Clara, CA, USA). Labeled DNA was cleaned up using MicroconTM YM-30 columns (Millipore, Billerica, MA, USA) and eluted in Tris-EDTA (TE) buffer. Cy5- and Cy3-labeled samples were combined in a single mixture and hybridized onto a 1x244K array for 40hs at 67°C. Microarrays were scanned using an Agilent microarray scanner and images analyzed with Agilent Feature Extraction software v10.7. Data were further analyzed by means of Agilent Genomic Workbench v5.0 software (Agilent Technologies, Santa Clara, CA, USA). The full list of CpG islands (CGIs) analyzed is based on the UCSC Genome Browser hg18, NCBI build 36.1, March 2006. Data were further analyzed according to the methodological approach conceived by Dr. Ravid Straussman and colleagues in 2009 [37].

### Pyrosequencing Analysis

Pyrosequencing experiments were aimed at quantitatively evaluate the methylation levels of the CpG-containing regions of MGMT and PDGFB genes. The assays were designed to investigate the same regions covered by MeDIP-Chip probes and encompassed 10 and 5 CpG sites for MGMT and PDGFB, respectively. The primers used were the following: MGMT: Fw 5′ – GTTTYGGATATGTTGGGATAG –3′, Rw 5′biotin – CRACCCAAACACTCACCAAA - 3′, Seq: 5′ – GATAGTTYGYGTTTTTAGAA –3′; PDGFB: Fw 5′- GGGGGGCGAAGGTAATGA –3′, Rw 5′biotin – CATAAATCGCTACTAAACGCTCTTCCTATCT - 3′, Seq: 5′ – ATGAAGAATTAGTTTTAGT –3′. PCR reactions were carried out using 20 ng of bisulphite-converted DNA from G144, G166 and CB660 cell lines in a final volume of 50 µl, with 10 pmol of forward and reverse primers, one of them being biotinylated. Quantitative DNA methylation analyses were performed using the Pyro Mark ID instrument in the PSQ HS 96 System (Biotage AB, Uppsala, Sweden), with the PyroGold SQA reagent kit (Biotage AB, Uppsala, Sweden) according to the manufacturer’s instructions. Raw data were analyzed using the Q-CpG software v1.0.9 (Biotage AB, Uppsala, Sweden), which calculates the ratio of converted C’s (T’s) to unconverted C’s at each CpG, giving the percentage of methylation. For each sample, the methylation value represents the mean between two independent PCR and pyrosequencing experiments.

### Gene Expression

Total RNA was extracted using the TRI Reagent solution (Applied Biosystems, Carlsbad, CA, USA). RNA was reverse-transcribed with SuperScript TM II Reverse Transcriptase (Life Technologies Italia, Monza, Italy) according to manufacturer’s instructions. For RT-PCR, amplifications were performed with 130 ng of RT product per reaction and 0.15 units of Platinum Taq DNA Polymerase High Fidelity (Life Technologies Italia, Monza, Italy), using a Mastercycler instrument (Eppendorf, Hamburg, Germany). PCR conditions used to detect constitutive HPRT and PTEN expressions were as follows: 3 minutes at 94°C, followed by 40 cycles at 94°C for 30 seconds, annealing at 62°C for 30 seconds, extension at 72°C for 1 minute, followed by a final extension at 72°C for 5 minutes. All the PCR products were electrophoresed on 1.6% agarose gels and stained with ethidium bromide. Sets of primers used to amplify HPRT and PTEN genes are listed below: PTEN 5′-CGAACTGGTGTAATGATATG -3′; 5′- CATGAACTTGTCTTCCCGTC -3′ (330 bp); HPRT 5′- AATTATGGACAGGACTGAACGTC -3′; 5′- CGTGGGGTCCTTTTCACCAGCAAG -3′ (388 bp).

Quantitative real-time PCR was performed using an Applied Biosystems 7500 Standard instrument (Applied Biosystem, Carlsbad, CA, USA) with gene-specific primers for WNT9A and WNT11genes (RT^2^ qPCR SYBR Green-based primers, SABioscience, Milan, Italy). Reactions were performed according to manufacturer’s guidelines.

### Bioinformatic Analysis

The Gene Ontology (GO) analysis was performed using GOstat software (http://gostat.wehi.edu.au/) [38], in order to identify possible enrichment of functional groups, related to “biological process”, in a specific input list of genes. GOstat software output file is a list of p-values for each GO term, estimating the probability that the observed counts could have occurred by chance. In order to limit the number of GO terms, a class should comprise more than five genes to be considered for further analysis [39]. GO terms were divided in cancer-relevant functional categories: 1. *cell cycle*; 2. *cell death and apoptosis*; 3. *response to external stimulus*; 4. *cytoskeleton organization*; 5. *cell signaling*; 6. *development & morphogenesis*; 7. *cell differentiation*; 8. *immune response*; 9. *cell motility*; 10. *metabolism;* 11. *transcription & gene expression*; 12. *intracellular transport*; 13. *DNA repair & chromatin remodeling*. Each category was associated to a frequency, which was calculated evaluating the ratio between the number of genes linked to a specific category and the total number of genes associated with at least one GO term. The pathway analysis was generated using the Ingenuity Pathway Analysis software (IPA, Ingenuity System, Redwood City, CA, USA, www.ingenuity.com). IPA software examines functional relationship within an input list of genes and identifies the pathways from the IPA library of canonical pathways that were most significantly associated with the dataset. The significance of the association between the data set and the canonical pathway was measured in 2 ways: 1) a ratio of the number of molecules from the data set that map to the pathway divided by the total number of molecules that map to the canonical pathway is displayed; 2) Fisher’s exact test was used to calculate a p-value determining the probability that the association between the genes in the data set and the canonical pathway is explained by chance alone. Network analysis displays regulatory relationships existing among the genes in the input dataset and then the involved networks are ranked by score. Networks are selected if their score is higher than 3, which means that there’s less than 1/1000 chance that the clustering would have occurred by chance.

### Statistical Analysis

Statistical analysis was carried out performing chi-square test, by means OpenEpi software v2.3.1, available on line at http://www.openepi.com/. The critical level of significance was set at p<0.05.

## Results

The stemness properties of the GSC lines were monitored during this study (although they were previously characterized [32], [33]), in order to ensure that the data obtained could be ascribed to the stem cell subpopulation of GBM. All the GSC lines retained a good proliferation rate, ability to form neurospheres (data not shown) and did not enter the differentiation program, as shown by the stable expression of stem cell markers (CD133 and nestin, Figure S1) in a rather constant percentage of cells.

### Cytogenomic Complexity of GSC Lines

Chromosome analysis was performed for all the six GSC lines and composite karyotypes (cp) were reconstructed. In addition, three out of six GSC lines (GBM2, G166 and GliNS2) were further analyzed by means of FISH, using a panel of whole chromosome painting probes. In this way, it was possible to identify recurrent structural abnormalities, which were not identified by conventional cytogenetic techniques (Table 1 and Figures S2, S3, and S4). Array CGH analysis was also performed for all cell lines (Table S2, S3, S4, S5, and S6) except for G144, whose genomic aberrations were previously reported [33]. The data discussed in this publication were deposited in NCBI's Gene Expression Omnibus [40] and are accessible through GEO Series accession number GSE41875.

**Table 1:** Chromosomal aberrations identified in the six GSC lines through QFQ-banding and FISH analysis.

	Cell line	GBM2	GBM7	G144	G166	G179	G179*	GliNS2
	**<ploidy>**	62∼83<3n>,XXY	67∼96<4n>,XXY	63∼95<4n>,XXYY	48∼59<2n>,XXXXY	64∼79<3n>,X,reaY	101∼117<5n>,XX,reaY	41∼47<2n>,XXY
**Chr.**	**X**	−[32]	−[19]	−[33]	−[50],–[14]	del(X)(p11.22)[100]	del(X)(p11.2)[100]	/
	**Y**	−[79]	−[19]	−[44]	−[61]	rea(Y)[67]	rea(Y)[100]	/
	**1**	+[29]	+[13], −[19]	−[28]	der(1)t(1;21)(q11;?)[100]	del(1)(p33)[50]	+[33]	inv(del(1)(p34.3))[79]
		der(1)t(1;9)t(p36.3;q13)[21]	del(1)(p36.1)[56]	der(1)t(1;?)(p13;?)[28]	rea(del(1)(p11))[89]		del(1)(p33)x2[50]	
		del(1)(p34)[26]	del(1)(p36.1)x2[13]	del(1)(q21)[11]				
			del(1)(p34)[19]					
			add(1)(p36.3)[13]					
	**2**	+[6], −[12]	+[50],del(2)(q?)[63]	−[22],del(2)(p11.2)[17]	rea(del(2)(p11))[89]	+[33]	–[67]	−[100],der(2)t(2;20)[100]
	**3**	−[12],del(3)(q13)[6]	−[31]	+[17], −[17]	+[82]	+[50]	+[83]	del(3)(p22?p25?)[97]
					rea(3)[7]			
	**4**	+[9], −[26]	+[13],del(4)(p12)[13]	−[72]	−[18]	−[83]	−[67]	−[14]
		der(4)t(3;4)(p21;p16)[17]						
	**5**	+[56],der(5)(q12;q13)[6]	+[19]	+[22], −[39],rea(5)[11]	+[82],++[7]	/	−[83]	der(5)t(2;5)(?;q35.3)[75]
					der(5)t(5;11)(p11;?)[84]			
	**6**	+[38],++[6], −[9]	−[44],–[19]	++[22], −[72]	+[11]	+[67]	+[33]	der(6)t(3;6)(?;q27)[65]
		del(6)(q14)[7],del(6)(q14)x2[9]			del(6)(q16.1)[89]			der(6)t(del(6)(q25.1);21)
		der(6)t(6;7)(q27;?)[62]						(p21?;p13)[100]
	**7**	+[47],++[44],del(7)(q31)[76]	+[25],++[56],+++[19]	+[39],++[44]	−[11]	++[83]	++[50]	+[97]
		del(7)(q31)x2[9]					del(7)(q11.21)[33]	
	**8**	+[44],++[12], −[9]	−[25]	−[56]	+[14]	+[67]	++[67]	−[14]
	**9**	+[62],del(9)(q11)[9]	−[31]	+[17], −[33]	+[7]	/	−[67]	/
		dic(9;del(9)(p11))(q34;q34)[6]		rea(9)(p?)[11]				
	**10**	+[21], −[21]	−[63]	+[44], −[22]	−[14]	–[67]	−[50]	−[17],del(10)(q21.3)[83]
	**11**	+[9], −[15]	−[25]	−[44],rea(11)[11]	+rea(11)[93]	/	−[67]	rea(del(11)(q13))[7]
				del(11)(p15)[11]				
	**12**	+[56], −[19],del(12)(p12)[24]	+[19], −[19],	+[22], −[33]	−[25]	+der(12)t(12;?)[100]	+[33], −[67]	+[7]
			del(12)(p12)[56]	del(12)(q?)[78]			der(12)t(12;?)[83]	
			del(12)(p12)x2[25]	der(12)t(12;?)(q11;?)[11]				
	**13**	−[88]	+[44]	+[11], −[72]	+[11], −[14]	/	–[83]	−[41]
			der(13;14)(q10;q10)x2[63]					
			der(13;14)(q10;q10)x3[31]					
	**14**	+[62], −[9]	+[19], −[19]	+[11], −[72]	−[39]	−[83]	−[50]	+[10], −[21]
	**15**	+[50], −[9]	−[31],–[31]	+[17], −[61]	+[21]	−[50]	+[67]	+[7], −[10]
	**16**	+[9], −[12]	+[13], −[19]	+[22], −[28]	+[82]	++[83]	++[83]	+[28],rea(16)[93]
	**17**	+[53],del(17)(p12)[9]	−[31],–[19]	+[33], −[28]	−[36]	/	+[33], −[50]	+[7]
		add(del(17)(p12))(q25)[6]			i(17)(q10)[96]			
	**18**	+[6], −[79],add(18)(q23)[9]	+[13], −[38]	−[28]	der(18)t(7;18)(?;p11)[90]	/	/	+[14],del(18)(p11.2)[79]
	**19**	+[12], −[18]	+[25],++[13]	+[44],++[33]	+[11], −[46]	/	/	−[10]
					der(19)t(19;22)(q13;?)[78]			
	**20**	+[29], −[21],–[15]	+[31],++[56]	−[44]	+[36],++[61]	/	−[67]	−[62]
		der(20)t(12;20)(q13;p11.2)[6]						
	**21**	+[59],++[29]	+[38]	+[17], −[28]	−[36]	−[83]	–[100]	−[24],der(21)t(6;21)[65]
	**22**	+[65],++[18]	−[19]	+[22], −[50]	−[93]	−[83]	−[83]	−[14]
	**mar**	1∼3	1∼2	1	1∼2	/	/	/

The aberrations identified and/or supported by FISH analysis are marked in red. -: loss of whole chromosome; +: gain of whole chromosome; ++: more than one sovrannumerary copy; –: loss of at least two copies of a chromosome;/: lack of alterations. All the numerical alterations are referred to the modal number of chromosomes for each cell line. Numbers in square brackets refer to percentages of each abnormality. From a minimum of 12 to a maximum of 34 metaphases were evaluated for each GSC line and the analysis was performed on at least 3 different passages.

**Figure 1:**
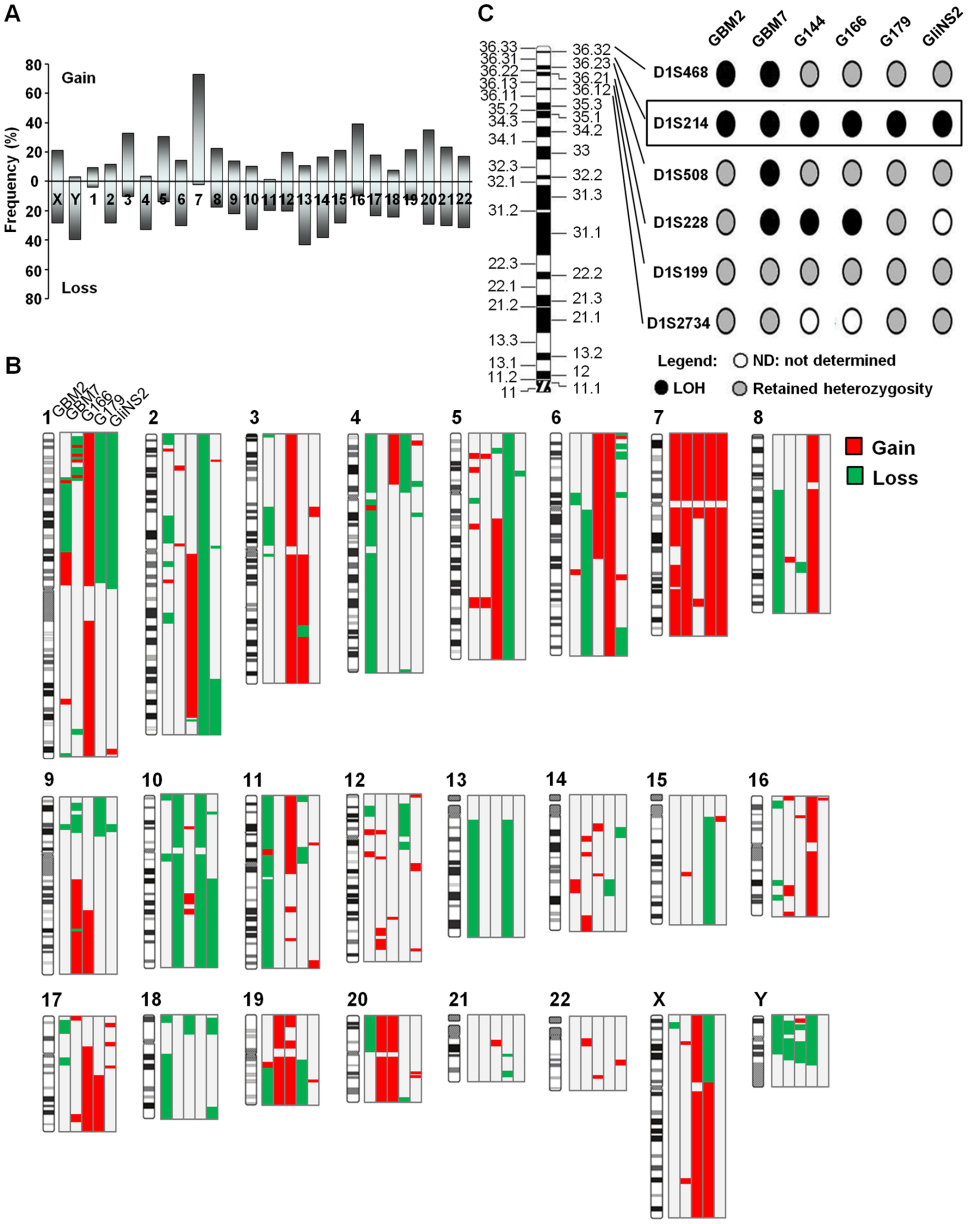
Cytogenomic profiles of GSCs. (A) Frequency of gains and losses of whole chromosomes in the six GSC lines analyzed by QFQ-banding. The frequencies of numerical aberrations specific for each chromosome were calculated from the total of the analyzed metaphases of the six cell lines and represented as mean values. (B) Composite array CGH profiles of GSC lines. (C) Detailed 1p LOH mapping of GSC lines. A common region of LOH was identified in all the six GSC lines, involving D1S214 microsatellite, located at 1p36.31 and highlighted by the square box.

All the clonal chromosomal abnormalities pointed out by this analysis are listed in Table 1. As expected, each cell line showed a certain degree of karyotype complexity which ranges from a high number of structural abnormalities, as the GBM2 cell line, to the presence of two subpopulations with a different modal number of chromosomes, as the G179 cell line (G179 and G179*). The modal number of chromosomes varied from near-diploid (G166, GliNS2), near-triploid (GBM2, G179), near-tetraploid (G144, GBM7), to near-pentaploid (G179*). All the chromosomes were involved in numerical alterations (Figure 1A): the most common (73% of the total analyzed metaphases) was gain of chromosome 7, with at least two supernumerary copies in four cell lines (GBM2, GBM7, G179 and G144, Table 1). Other commonly observed numerical changes were: loss of chromosome 13 (43%); loss or gain of chromosome X (28% or 21% of cases, respectively); loss of chromosome Y (39%); loss of chromosome 10 (in three out of six cell lines, frequency of 32%). A total of 59 different clonal chromosomal aberrations were found among the 6 cell lines. Chromosome 1 was the most involved in structural abnormalities: the ever present deletions in 1p36-1p33 were further accompanied by inversions or unbalanced translocations (Table 1 and Figures S2, S3, and S4). Also chromosomes 18, 11 (three out of six cell lines), 12 (four out of six cell lines) were frequently damaged by structural abnormalities. Finally, the long arm of chromosome 6 was affected by loss of genomic material in two cell lines (GBM2 and G166) or by two translocations, involving chromosome 7 (GBM2) or 3 (GliNS2). Lastly, by conventional cytogenetic techniques, it was possible to observe the presence of *double minutes,* which should not be included in the count of the number of chromosomes. The molecular karyotypes showed some common genomic features of GBM, such as complete loss (nullisomy) of 9p21.3 locus (GBM2, GBM7, G179 and GliNS2), containing CDKN2A and CDKN2B genes (Figure 1B and Tables S2, S3, S4, S5, and S6). Complete or nearly complete gain of chromosome 7 was evidenced in the same GSC lines, while for G166 cells gain of 7p22.3-q11.2, including EGFR, was observed (Figure 1B and Table S4). Loss of whole chromosome 10 in GBM7 and G179 cell lines led to the inevitable absence of PTEN and DMBT1 genes and the same alteration was obtained in GliNS2 cells through the loss of 10q21.3-q26.3 region (Table S6). Accordingly, loss of PTEN locus (10q23.31) resulted in the lack of detectable PTEN transcriptional expression in G179 and GliNS2 lines and slight expression in GBM7 cells. Considering the absence of genomic alterations at PTEN locus in GBM2 and G166 cell lines, the divergences in gene expression should be ascribed to differences in the methylation levels of PTEN promoter region: G166 cells revealed PTEN expression and lack of promoter methylation, while GBM2 cells displayed no PTEN expression and promoter methylation (Figure S5). Loss of whole chromosome 13, containing RB1 gene, was evidenced in 86% of GBM2 and 29% of G179 cells (Table S2 and S5, respectively). Several CNAs affected chromosome 1: whole p-arm loss nearly in 50% of G179 cells; 1p36-p34 loss in GBM7 and GliNS2 cell lines; 1q21.1-q32.2 gain in G166 (almost 90% of cells); in particular, gain of 1q32.1 locus, containing MDM4 gene, was found also in GBM2 cells. Other aberrations were: gain of 20p and 20q in GBM7 and G166 cells; loss of chromosome Y in four cell lines. In addition, “private” alterations were evidenced, such as the gain of whole chromosome X in G166 line, the amplification of 4q12, containing PDGFR and the loss of TP53 locus (17p13.2-p13.1) in almost 60% of cells in GBM2 line.

### Loss of Heterozygosity (LOH) Analysis

LOH analysis was performed using a panel of microsatellites, spanning on regions mainly involved in GBM pathogenesis. Microsatellites for chromosomes 10 and 13 were found mainly heterozygous (data not shown). Microsatellite analysis of chromosome 1p revealed segmental LOH in all the cell lines (Figure 1C). Discontinuous loss was defined as interstitial or small terminal deletions at one or more loci, with retention of heterozygosity at the proximal end of the evaluated region, with or without retention of heterozygosity at the distal end [41]. Precisely, LOH was discontinuous in G144 and G166 cell lines; interstitial in G179 and GliNS2 cell lines; and telomeric in GBM2 and GBM7. The D1S214 microsatellite in 1p36.31 was deleted in all the six GSCs analyzed and it maps in the open reading frame of Calmodulin binding transcription activator 1 (CAMTA1) gene.

### Epigenomic Landscape of GSC Lines

**Figure 2:**
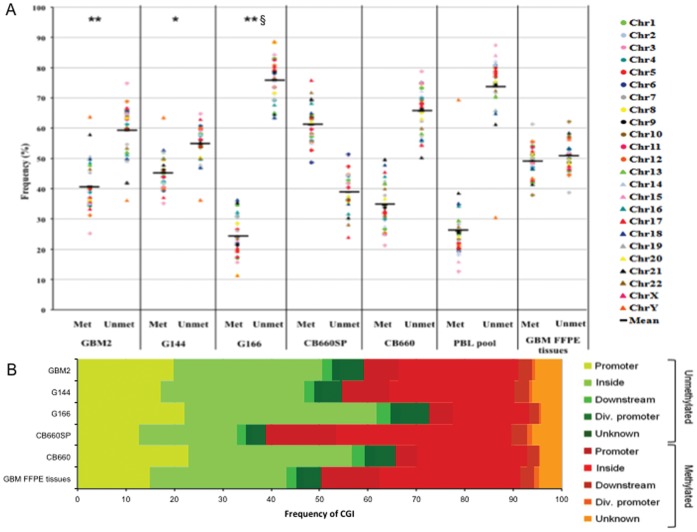
Methylation profiles. (A) Frequency of methylation and unmethylation of CGIs for each sample. The methylation status for each chromosome is reported and global genomic methylation percentages are displayed as the mean values of all chromosomes values. GSCs vs. CB660SP *p<0.05, **p<0.01, GSCs vs. GBM FFPE tissues §p<0.01, Chi-square test. Abbreviation: Met, methylation; Unmet, unmethylation. (B) Distribution of methylated and unmethylated CGIs among the different functional genomic regions.

As DNA methylation is a key component of genome regulation in normal and cancer tissues, we evaluated the methylation status of three GSC lines. The array platform used in this study covers 27800 CGIs of the human genome [42] and all the data (percentages and frequencies) are referred to the total number of CGIs included in the array. Raw data were processed as described in the material and methods section and they were deposited in the NCBI's Gene Expression Omnibus [40] and are accessible through GEO Series accession number GSE41824. GSC data were compared with methylation levels of two foetal neural stem cell (NSC) lines, which were considered as matching normal control cells: CB660 derived from human foetal forebrain, and CB660SP isolated from human foetal spinal cord [34]. Additionally, data were compared with DNA methylation status of an equimolar pool of genomic DNA from GBM FFPE tissues and an equimolar amount of genomic DNA from peripheral blood lymphocytes of six healthy male donors (PBL pool), used an unrelated type of tissue. Results were plotted on a chart showing CGI methylation or unmethylation frequencies and mean genomic values were calculated (Figure 2A). Considering the global DNA methylation data, an overall CGI hypomethylation of GSC lines was noticed compared to foetal NSCs derived from the spinal cord, CB660SP (CGI methylation was lower than 50%). On the other hand, the global CGI methylation percentages of GSCs were similar to CGI methylation levels of foetal forebrain NSCs that showed half the CGI methylation content in comparison with CB660SP cells (35.4% vs. 61.1%, respectively). GBM FFPE tissues showed a CGI methylation level of 49%, which differs significantly from the G166 one, but not from those of GBM2 and G144 cell lines. PBL pool exhibited low level of methylation (26.3% of methylated CGIs); however, this percentage has already been reported in literature [43]. Going into deep the distribution of CGI methylation across the genomic regions was analyzed. Even if, the overall CGI methylation levels of GSCs, FFPE GBM tissues and CB660 forebrain NSCs were similar, almost a doubling in the CGI methylation percentages in promoter and divergent promoter regions was noticed in GBM2, G144 and GBM FFPE tissues related to CB660 cells (Figure 2B and Table S7).

**Figure 3:**
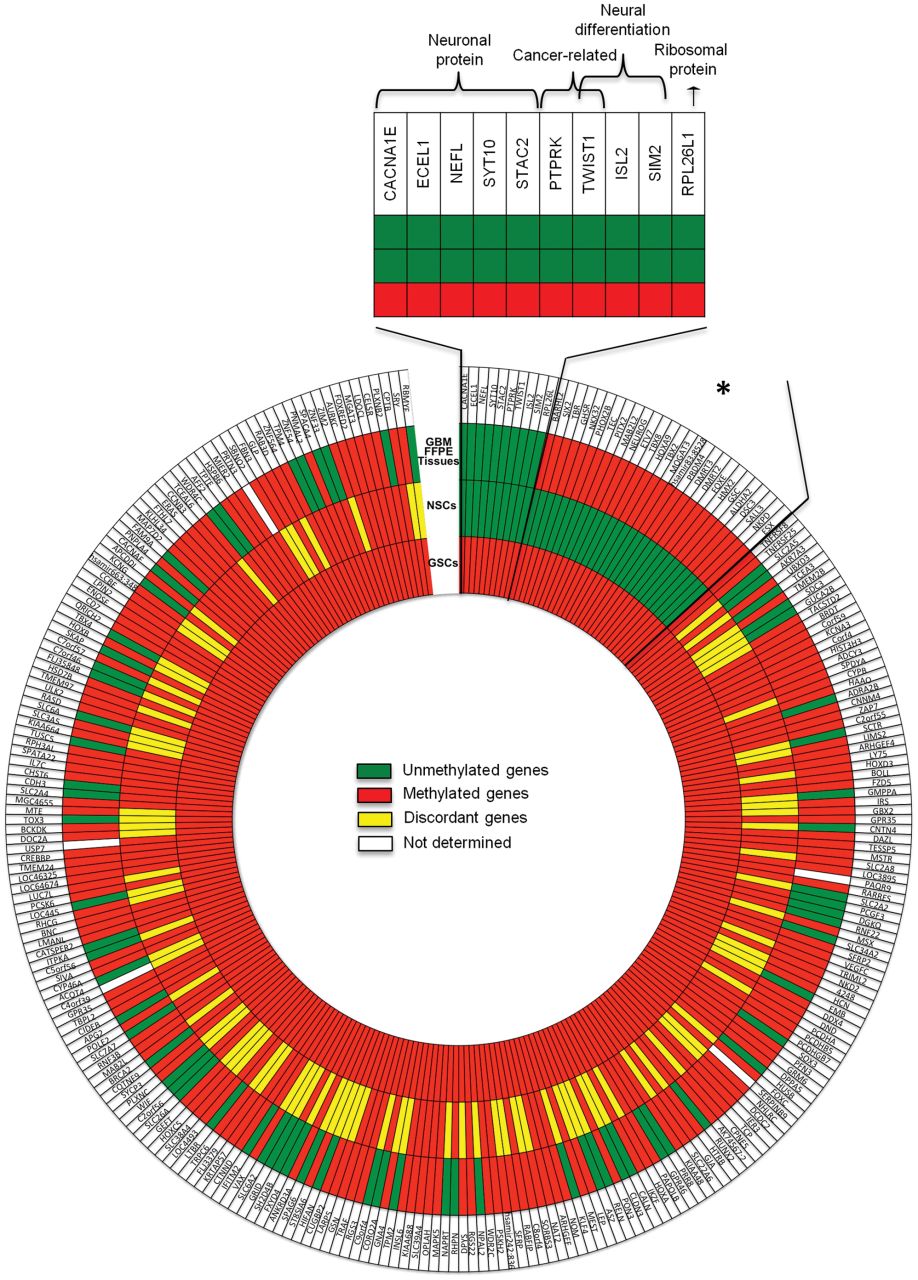
GSC epigenetic signature. Epigenetic comparison between GSC, NSC and GBM FFPE tissue methylation profiles. The inner circle shows the 378 shared methylated genes in GSC lines, while the middle circle points out 37/378 genes that were unmethylated in NSC lines. The external circle displays the methylation status in GBM FFPE tissues. Note that 10 of the 37 specifically methylated genes in the GSC lines and unmethylated in foetal NSC lines were unmethylated in GBM FFPE tissues, representing the GSC epigenetic signature. The asterisk identifies 27 “cancer *de novo* methylated genes” in GSC and GBM FFPE tissues vs. foetal NSCs (see also Table S9A).

As the methylation or unmethylation of promoters is associated to a specific biological effect (repressing or allowing transcription, respectively), whereas the methylation of other functional genomic regions remains controversial [44], [45], we deepened our analysis only for promoter regions. We investigated the methylation status of selected genes, whose hypermethylation is specifically associated to GBM: RASSF1A, CDKN2A, MGMT, RB1, CDH1 and EMP3 (Table S8) [46]. These genes showed a heterogeneous pattern of methylation among the GSC lines and were mainly methylated in the GBM FFPE tissues rather than in the GSC lines. Thus, in order to identify a “GSC-specifically methylated genes” signature we compared the CGI methylation status of GSC lines, GBM FFPE tissues and foetal NSC data. We identified 27 gene promoters methylated in all three GSC lines and FFPE GBM tissues and unmethylated in both the foetal NSC lines (Table S9A). Moreover, this comparison also pointed out 10 genes exclusively *de novo* methylated in GSC lines and not in GBM FFPE tissues related to NSCs, delineating a subgroup of genes exclusively methylated in GSCs (Figure 3 and Table S9B).

### Validation of MeDIP-Chip Analysis

In order to validate MeDIP-Chip results, pyrosequencing of two genes (MGMT and PDGFB) was performed on G144, G166 and CB660 cell lines. MeDIP-Chip experiments showed that MGMT promoter was methylated in these three cell lines. We scored as methylated the MGMT promoter of G144 and CB660 lines, whereas G166 displayed an intermediate methylation level (Figure S6A).

Regarding the methylation status of PDGFB promoter, the pyrosequencing analysis confirmed the MeDIP-Chip data: in particular, G166 and CB660 resulted unmethylated for this region, while G144 was methylated (Figure S6B).

Moreover, we checked the correlation between promoter methylation, identified by array analysis, and gene expression (WNT9A and WNT11 genes). In both cases an increased expression was evidenced in unmethylated gene promoters compared with methylated ones (Figure S7).

### Functional Annotation and Pathway Analysis

**Figure 4:**
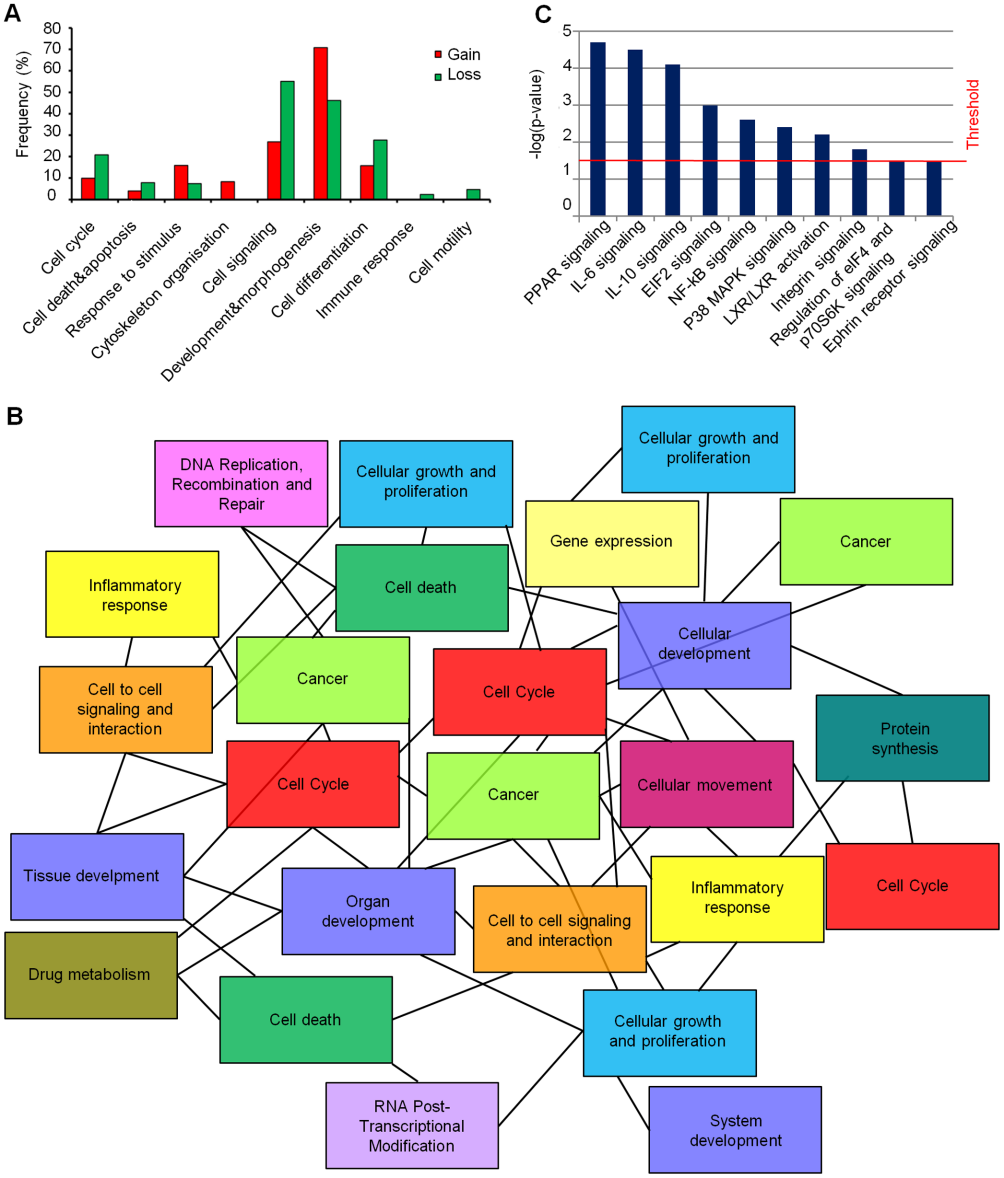
Functional characterization of cytogenomic landscapes. (A) Categories of genes determined by GO analysis and included in gain and loss regions. Each category is associated to a percentage of frequency which was calculated on the ratio between the number of genes associated to a specific category and the total number of genes associated to at least one GO term. (B) Tree topology of overlapping network established using IPA software. Genes in new “exclusive” gain and loss regions identified in GSCs profiles of aCGH were assigned to gene networks which were strictly interconnected one to each other and revealed cancer-relevant annotations. Different genes can be grouped in several networks, underlying the same mechanism (i.e. cancer or cell cycle). (C) New ‘exclusive’ CNA region-associated pathways. Each pathway is associated with a p-value (calculated by Ingenuity Pathway Analysis, IPA, software), which represents the probability that such association could have occurred by chance.

Genome-wide data were analyzed through GOstat and IPA software in order to identify biological functions and pathways related to input gene lists, respectively. Cancer-related GO terms were grouped in different functional categories, as described in the materials and methods section. Each category was scored based on its own percentage of genes belonging to that specific category [47] and normalized to the total number of genes. *Cell signaling* and *development and morphogenesis* were the most represented biological functions in gain and loss regions, which underlie a de-regulation of genes related to these categories by amplification or deletion of genomic regions (Figure 4A). Moreover, other categories resulted affected by CNAs, i.e. *cell cycle*, *apoptosis*, *cell differentiation*, *response to stimulus* and *cytoskeleton organization*. Furthermore, even if at lower frequencies, *cell motility* and *immune response* categories were associated with deleted regions (Figure 4A).

In order to define a kind of stem-cell genomic signature of GBM, we compared aCGH data from GSC lines to CNAs derived from 430 genomic profiles described in literature studies [20], [48]–[51] obtained from both surgical specimens and serum-cultured cell lines derived from GBM, so they may be considered as a bulk tumor genomic signature. Although several new “exclusive” affected regions emerged from our GSC line profiles (Table S10), unfortunately none of these regions was shared among them. Anyway, we analyzed through IPA software the genes mapping in these “exclusive” affected regions in order to identify shared networks and pathways. Specifically, genes located in these apparently divergent and “exclusive” affected regions were strictly associated into interconnected networks, describing a strong functional relationship converging towards a common mechanism of de-regulation between the GSC lines (Figure 4B). In Figure 4C were reported the specific pathways affected by the “exclusive” CNA regions and identified by IPA analysis (see also Table S11).

**Figure 5:**
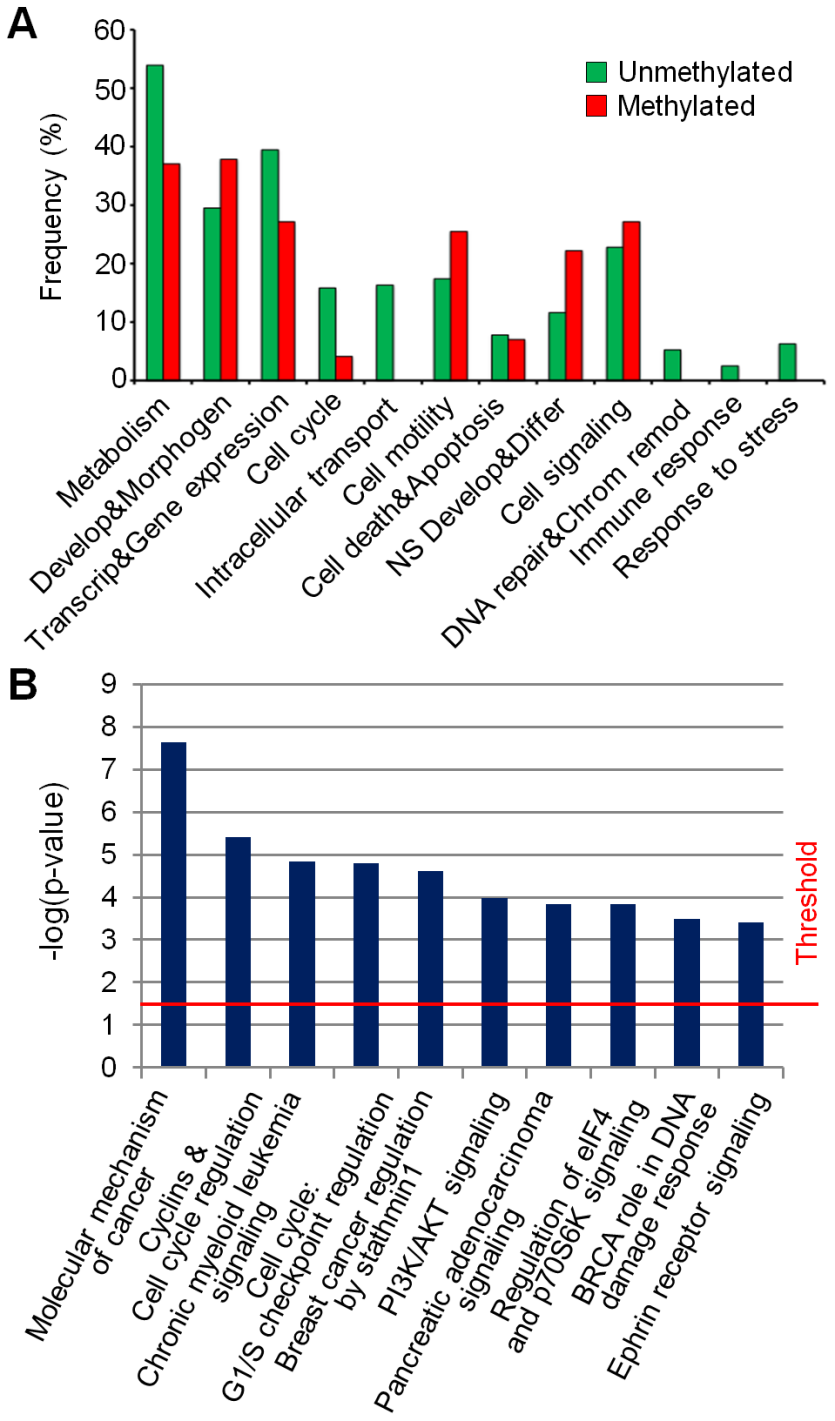
The GSCs’ methylation profiles evidence the functional impairment of cell development and differentiation processes. (A) Functional annotation analysis of commonly methylated or unmethylated gene promoters in all the three GSC lines (GBM2, G144 and G166), performed using GOstat software. The graph shows the percentage (y-axis) of each category compared to totally annotated genes. (B) Top 10 pathways influenced by DNA methylation pattern in GSCs. A p-value (calculated by the Ingenuity Pathway Analysis, IPA, software) is associated to each pathway; this value represents the probability that such association could have occurred by chance.

Similarly, DNA methylation data were analyzed through these bioinformatic tools. Firstly, the analysis of gene promoters with the same methylation pattern among GSC lines showed an enrichment of terms related to the *metabolism* category, with a prevalence of unmethylated gene promoters (Figure 5A). Increased levels of unmethylation were found in other two categories: *transcription & gene expression*, which could lead to the activation of cancer-related genes, and in *cell cycle*, showing the de-regulation of cell proliferation in GSCs. On the other hand, GSCs showed a prevalence of methylated terms associated to *development & morphogenesis* and *nervous system development & differentiation,* showing an impairment of the developmental and differentiation processes. *Cell death & apoptosis* showed a balance between methylated and unmethylated gene promoters, thus epigenetic changes in these genes might act in order to maintain the malignant “homeostasis” of tumor cells. Conversely, four categories were involved only in unmethylated gene promoters (*intracellular transport*, *DNA repair and chromatin remodeling*, *immune response* and *response to stress)* perhaps increasing the potential malignant phenotype of GSCs. The analysis of gene promoters with the same methylation pattern among GSC lines through IPA software revealed the involvement of several cancer-related pathways (Figure 5B, Table S12). Curiously, two pathways had already been identified from the previous analysis of “exclusive” CNA regions (*regulation of eIF4 and p70S6K signaling* and *ephrin receptor signaling)*, indicating that genomic and epigenomic alterations converge in the same direction.

Secondarily, the analysis focused on 27 gene promoters which constitute the ‘cancer *de novo* methylated genes’, as they were aberrantly methylated in GSC lines and GBM FFPE tissues compared with foetal NSC lines (Figure 3 and Table S9A). A strong enrichment of neurodevelopmental process related genes was evidenced. To achieve a deeper insight into the mechanisms underlying DNA methylation in GBM, we evaluated if these 27 cancer-specifically methylated genes were targeted by Polycomb repressive complex 2 (PRC2) in embryonic stem (ES) cells [52]. We found that 13/27 (48.1%) genes were targeted by Suz12 protein, a subunit of PRC2 and such enrichment was statistically significant (10% of genes are marked by PRC2 complex in ES cells, Fisher’s exact test, p<0.001) [52]. Lastly, the comparison of CGI methylation in GSCs, total GBM tissues (FFPE GBM pool) and foetal NSCs allowed the identification of 10 genes exclusively methylated in the stem cell subpopulation of GBM (Figure 3 and Table S9B). Moreover, these genes showed prevalence in the neural determination and differentiation processes and 3 genes (TWIST1, ISL2 and SIM2) were targeted by the PRC2 in ES cells.

## Discussion

### Towards the Delineation of a Cytogenomic and Epigenomic “Signatures” Specific for GSCs?

A fundamental issue regarding GSC is the uncertainty of GSC markers, to the extent that the derivation of robust signatures describing the GSC subpopulations has become almost the Holy Grail of research. Indeed, tumors may harbor multiple phenotypically or genetically distinct CSCs, as we verified in GBM [25], thus it will be necessary to target not only all the GSC subsets within a tumor, but at the same time the non-tumorigenic cells, for their ability to revert to a tumorigenic state [15]. The recent “back to Darwin” model for cancer propagation, suggested by Greaves in 2010, assumes cells with variable self-renewal potential or “stem cells” as the genetically diverse units of evolutionary selection [19]. Taking this model into consideration, we applied an integrated analysis on six GSC lines, considering them in their entirety from the genetic and epigenetic point of view in order to achieve a comprehensive insight into the cytogenomic and epigenomic landscapes of GBM. The data collected in this work emphasize the importance of studying GBM, but the observations can be extended to other types of cancer, by analyzing them entirely at different molecular levels, like the layers of an onion. Indeed, we found several canonical cytogenetic alterations of GBM, such as: i) partial or whole gain of chromosome 7, leading to gain of EGFR gene (7p11.2), identified in all six GSC lines and associated with approximately 40% of GBMs [20] (Table 1, Figure 1A and 1B); ii) loss of chromosome 13, associated with RB1 gene loss, a tumor suppressor gene localized at 13q14 and deleted in 30% of GBM cases [53]; iii) nullisomy of 9p21 locus, including CDKN2A and CDKN2B genes, is linked to a poor prognosis, as the lack of these negative regulators of cell cycle affects p53 and Rb pathways as well [54]; iv) loss of chromosome 10, encompassing PTEN gene at 10q23. Considering this last example, anyone can appreciate the usefulness of our multi-level analysis. Indeed, although 2/5 cell lines (GBM2 and G166) showed no damage in PTEN pathway at least at the cytogenetic level, PTEN expression was identified in 2/5 cell lines (GBM7 and G166) (Figure S5). So, where cytogenetics is unable to explain, cytogenomics and epigenomics come to rescue, because the low level of PTEN expression in GBM7 can be ascribed to the mosaic level of loss of this region (58% of cells), while the lack of expression in GBM2 cell line can be caused by the hyper-methylation of PTEN promoter (Figure S5). Another common alteration (30% of astrocytomas) is 1p deletion [55], identified in all the cell lines analyzed, both at the cytogenetic and genomic levels (Table 1 and Figure 1B), suggesting the presence of a tumor suppressor gene [56]. Although this region is quite large (1pter-p32) and the cytogenetic breakpoints are variable, we identified a minimal deleted region (MDR) at 1p36.31 in all six GSC lines by means of microsatellite analysis, including CAMTA1 gene, (Figure 1C). This MDR overlaps the 1p MDRs described in gliomas [41], [56] and neuroblastomas [57]. CAMTA1 gene was found down-regulated in CSCs compared with NSCs [58], meanwhile its up-regulation reduced colony formation in GBM cells both *in vitro* and *in vivo*, so its functional haploinsufficiency seems to be associated with a proliferative advantage [59]. Ultimately not considering all the well-known CNAs relating to GBM (a kind of bulk tumor genomic-signature), our cytogenetic-genomic analysis didn’t evidence any new alteration shared among the six GSC lines, defeating our efforts to delineate a GSC genomic-signature. However, on one hand our data confirm a role of driver mutations for the CNAs included in the GBM genomic-signature, on the other it is not obvious that the new CNAs are passenger mutations, as they may be necessary for tumor progression specific for the individual patient. If we stopped at this level of observation it would be like looking closely at the crop circles, losing the overall design that can be appreciated only through an aerial view. In fact, at a first level of complexity each tumor can perturb individual genes via multiple mechanisms (see above the example of PTEN gene) and a pathway can be damaged at different levels (genomic, genetic and epigenetic). On the other hand the picture becomes much more complicated when one considers that cellular networks contain functional modules and that tumors target specific modules critical to their growth. Through our approach, we were able to demonstrate that different combinations of perturbed genes can incapacitate each module. The same concept has been proposed using a network-based approach by Cerami et al (2010) [60]. This new overview could a have huge importance in therapy as it could explain the CSC resistance to targeted inhibition. Interestingly, as reported for other types of cancers [61], it has been recently demonstrated that GBM therapeutic resistance to EGFR inhibitors may be explained by compensatory activation of EGFR-related family members (ERBB2, ERBB3), and therefore simultaneous shutdown of multiple ERBB family members may be required for more effective GBM therapy [62]. For example, we decided to better investigate the cytogenomic and epigenomic states of IFNB1-STAT3 signaling. Signal transducer and activator of transcription 3 (STAT3) activation is crucial in the maintenance of GSCs: it is upregulated in GBM and has an anti-apoptotic role [63]. Upstream of STAT3 is interleukin (IL)-6 and IL-6 receptors are preferentially expressed in GSCs [64]. Moreover, recent evidence *in vitro* have also documented a role for IFNB1 that reduced GSC proliferation via STAT3-mediated differentiation into oligodendrocytes [65]. In addition, IFNB1 *in vitro* treatment reduced levels of miR-21, one of the most commonly upregulated miRNAs in glioma, via STAT3 activation. Looking at our data, 4 over 5 GSC lines had loss at 9p21, where IFNB1 is localized. G166 line didn’t loose IFNB1, but 85% of cells showed gain at 17p11.2-q25.3, resulting in the gain of the downstream signaling genes STAT3 and miR-21. Curiously, the same G166 line had 90% of cells that carried a gain at 1q21, where IL-6R-alfa is located. In this way, although with different mechanisms, the signaling is damaged, because on one hand the loss of IFNB1 might cause the non-activation of STAT3 and consequently the non-inhibition of miR-21, leading to the block of differentiation and apoptosis. On the other hand gain of IL-6R, STAT3 and miR-21 could lead to the same effect, as endogenous IFNB1 may be insufficient to ensure the functionality of the pathway. Ultimately, beyond the differences that can create apparent heterogeneity of alterations in GSC lines, there’s a sort of selective force acting on them in order to converge towards the impairment of cell development and differentiation processes.

### Functional Analysis Confirmed the Impairment of GSC Developmental and Differentiation Processes

Functional annotation analysis of gene set identified in CNA regions of GSCs confirmed an impairment of cellular development and differentiation processes. Indeed, developmental regulators may support the malignant phenotype and the stem-like cell properties, including robust self-renewal potential, shifting the balance towards the maintenance of an undifferentiated phenotype [66], [67]. We identified some functional annotations specific for the stem cell properties of GBM cells. Among the highly ranked pathways can be found: *NF-κB signaling*; *inflammatory cytokines signaling pathways* (IL-10 and IL-6); *integrin and ephrin receptor signaling* pathways. Thus, genomic analysis may help in the identification of specific signaling pathways, which play essential functional roles in cancer stem-like cells [68].

Epigenomic modifications, such as DNA methylation, are an integral part of the molecular determinants which contribute to malignancy [69]. The comparison between CNAs and DNA methylation patterns at promoter regions in GSC lines showed that aberrant methylation occurred both in regions affected by CNAs and in regions not affected by these alterations. Thus, methylation changes in gene promoters seemed to be unrelated to aberrant copy number [70]. Our data show, for the first time to our knowledge, the genome-wide methylation profiles of the stem cell subpopulation of GBM. Analyzing the global GCI methylation content, GCSs and GBM FFPE tissues showed GCI hypomethylation compared to foetal NSCs isolated from the spinal cord (Figure 2). Anyway, forebrain NSCs could be a more appropriate control tissue for GSCs, considering their origin and the molecular pathways involved in the two cell populations [71]. Thus, even if global CGI methylation didn’t seem to differ severely from one cell type to another, a deeper insight actually revealed a doubling of CGI methylation in promoter regions of GSCs and GBM FFPE tissues compared with CB660 cells. Promoter hypermethylation is frequently noticed in cancer as it contributes to tumorigenesis through the downregulation of tumor suppressor genes or genes normally involved in cell development and differentiation [46]. So, at the epigenetic level the differences in the methylation status of promoter regions may indicate a sort of “master” alteration that could influence the differentiation properties of GSCs. Anyway, DNA methylation profiles were heterogeneous, preventing the detection of a univocal behavior, so further insights will be needed in order to clarify this issue.

The specific pattern of promoter methylation shared by the three GSC lines enabled the identification of key biological functions related to the methylation profiles. *Metabolism* was the most enriched function, as cancer cells may require fast cellular turnover, according to their high replicative phenotype [31]. *Transcription* and *nervous system development and differentiation* were the other top ranking categories, suggesting that neurodevelopmental genes may be crucial for the full-stem like phenotype of glioma cells [72].

Pathway analysis showed that only two molecular signaling were shared between genomic and methylation profiles. Genetic and epigenetic changes are generally mutually exclusive in a given tumor [73] and they act synergistically on several signaling pathways, contributing together to tumorigenesis [74].

The analysis of the methylation status of GSC lines and GBM FFPE tissues in comparison with NSCs identified 27 “cancer *de novo* methylated genes” (Table S9A and Figure 3). These genes were found mainly involved in *transcription* and *cellular neurodevelopmental* processes. Moreover, 48.1% of these genes were identified as Polycomb group targeted (PCGT) genes in ES cells [52] and similar data were previously reported in a large series of GBMs [75]. In this study the differences between GSCs and NSCs were highlighted, because they could indicate which errors may deviate cancer stem cells from the correct program of differentiation. This analysis pointed out 10 more genes exclusively and aberrantly *de novo* methylated in GSC lines compared with NSC lines. These genes could be considered a sort of stem cell CGI hypermethylation signature associated to GBM (Table S9B and Figure 3). In particular, these genes encode for structural neuronal (CACNA1E, ECEL1, NEFL, SYT10, STAC2) and neural differentiating proteins (ISL2, SIM2, TWIST1), cancer-related factors (PTPRK and TWIST1) and a ribosomal protein (RPL26L1). Furthermore, 3 out of 10 were epigenetically regulated by PRC2 in ES cells (TWIST1, ISL2 and SIM2), suggesting an important role of methylation events on these genes affecting cell differentiation processes and cancer [76]–[78]. Thus, considering all these data the Polycomb connection should be strongly supported. In ES cells, PcG (Polycomb group) proteins reversibly repress genes encoding transcription factors involved in development and differentiation, forming the so called “bivalent domains” [79], [80]. *De novo* methylation at promoter regions of these genes may lock cells in a stem cell phenotype and promote aberrant clonal expansion [81], [82] in GBM development [81]. Indeed, aberrant methylated genes in GSCs were highly enriched in terms related to *nervous system development* and *neurogenesis*. Thus, an accumulation of a subpopulation of cells unable to differentiate can occur and novel transforming aberrations (both genetic and epigenetic) can be further acquired [83]. The involvement of PCGT genes in many types of cancers stresses the importance of this developmental gene class in tumorigenesis, highlighting a kind of conserved aberrant methylation pattern in cancer cells which might be considered a sort of epigenetic hallmark [84], [85].

In conclusion, the investigation of multiple levels by genome-wide profiles is a valuable tool to identify the molecular landscapes specific for the stem-cell counterpart in GBM and other types of cancers. This study pointed out the aberrant methylation of cancer and stem cell relevant genes associated with GBM and thus this analysis could be the starting point for future works in order to understand stem cell properties of GBM cancer cells. Moreover, the impairment of cell development and differentiation of GSCs stresses the importance of a differentiation-inducing therapy in the eradication of the stem cell subpopulation in GBM.

## Supporting Information

Figure S1 **Selected representative images of GSC immunofluorescence in standard growth conditions.**
(DOC)

Figure S2 **Panel of GBM2 chromosomal abnormalities identified through FISH analysis.**
(DOC)

Figure S3 **Panel of G166 chromosomal abnormalities identified through FISH analysis.**
(DOC)

Figure S4 **Panel of GliNS2 chromosomal abnormalities identified through FISH analysis.**
(DOC)

Figure S5 **RT-PCR performed on RNA obtained from GBM2, G166, G179, GliSN2 and GBM7 cells.**
(DOC)

Figure S6 **Quantitative CpG methylation analysis of A) MGMT and B) PDGFB promoters.**
(DOC)

Figure S7 **Real-time PCR data and promoter methylation of selected genes.**
(DOC)

Table S1 List of STS markers.
(DOC)

Table S2 List of CNAs and mosaic level in GBM2 cell line.
(DOC)

Table S3 List of CNAs and mosaic level in GBM7 cell line.
(DOC)

Table S4 List of CNAs and mosaic level in G166 cell line.
(DOC)

Table S5 List of CNAs and mosaic level in G179 cell line.
(DOC)

Table S6 List of CNAs and mosaic level in GliNS2 cell line.
(DOC)

Table S7 Percentages of methylated and unmethylated CGIs in GSCs, foetal NSCs, PBL pool and GBM FFPE tissues, classified in the different functional genomic regions.
(DOC)

Table S8 Methylation status gene promoters associated to GBM pathogenesis.
(DOC)

Table S9 A. Cancer *de novo* methylated genes: GSC and GBM FFPE tissues vs. foetal NSCs. B. *De novo* aberrantly methylated genes in GSC lines.
(DOC)

Table S10 New ‘exclusive’ CNAs of GSC lines identified through a comparison with literature data.
(DOC)

Table S11 Top 10 pathways associated to new ‘exclusive’ CNA regions.
(DOC)

Table S12 List of Top 10 pathways influenced by DNA methylation pattern in GSCs.
(DOC)
