# Supplementary material for: Delineating the Cytogenomic and Epigenomic Landscapes of Glioma Stem Cell Lines
Source: PLoS One. 2013 Feb 28;8(2):e57462. doi: 10.1371/journal.pone.0057462 (PMC3585345; doi:10.1371/journal.pone.0057462)

***Figure S1. Selected representative images of GSC immunofluorescence in standard growth conditions.*** Cells were spotted on glass slides using cytospin. Each specific maker is in green; phalloidin is in blue and propidium iodide in red. GSC cultures contained numerous immunoreactive cells for CD133 and nestin. Scale bar=100μm.


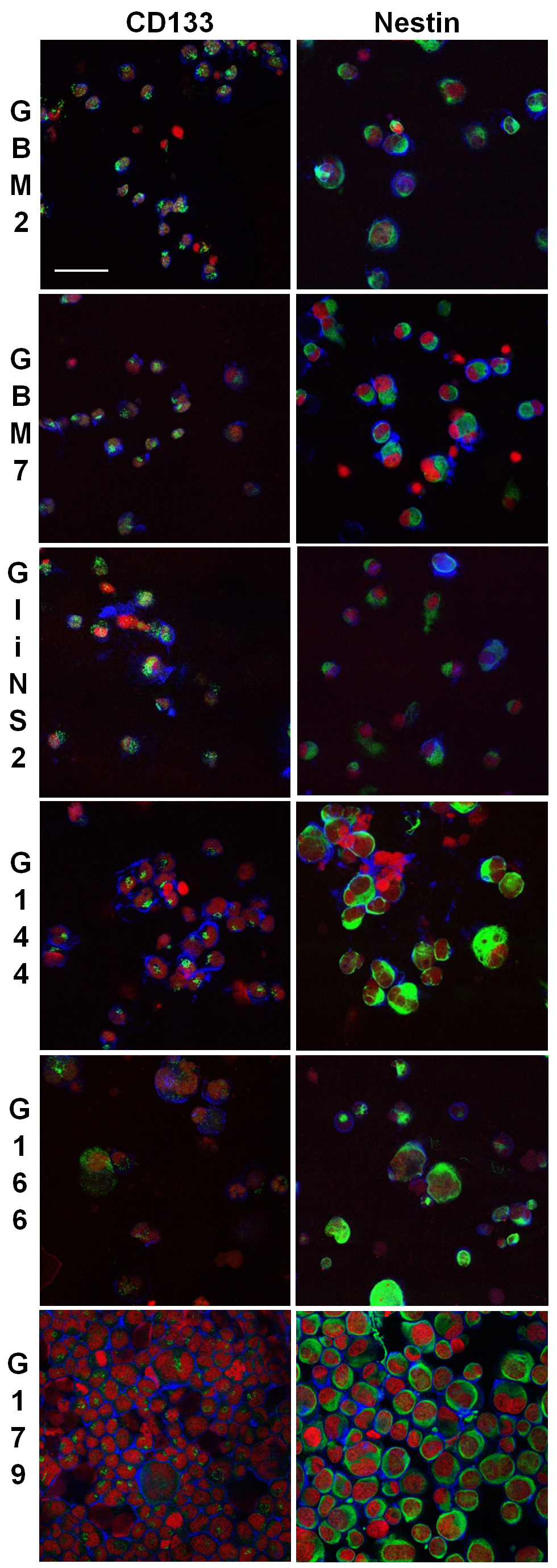

Supplement: Figure S1 — Selected representative images of GSC immunofluorescence in standard growth conditions. (DOC) [file pone.0057462.s001.doc]
