# Supplementary material for: Delineating the Cytogenomic and Epigenomic Landscapes of Glioma Stem Cell Lines
Source: PLoS One. 2013 Feb 28;8(2):e57462. doi: 10.1371/journal.pone.0057462 (PMC3585345; doi:10.1371/journal.pone.0057462)

***Figure S2. Panel of GBM2 chromosomal abnormalities identified through FISH analysis.*** Each aberration is described by means of QFQ-banded chromosomes and the corresponding FISH results. A. der(1)t(1;9)(p36.3;q13), B. der(4)t(3;4)(p21;p16), C. del(6)(q14), D. der(6)t(6;7)(q27;?).


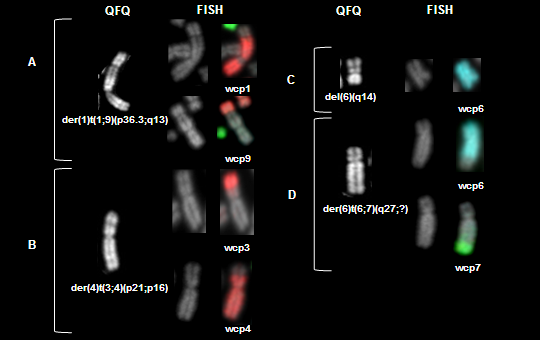

Supplement: Figure S2 — Panel of GBM2 chromosomal abnormalities identified through FISH analysis. (DOC) [file pone.0057462.s002.doc]
