# Supplementary material for: Delineating the Cytogenomic and Epigenomic Landscapes of Glioma Stem Cell Lines
Source: PLoS One. 2013 Feb 28;8(2):e57462. doi: 10.1371/journal.pone.0057462 (PMC3585345; doi:10.1371/journal.pone.0057462)

***Figure S3. Panel of G166 chromosomal abnormalities identified through FISH analysis.*** Each aberration is described by means of QFQ-banded chromosomes and the corresponding FISH results. A. der(1)t(1;21)(q11;?), B. rea(del(1)(p11)), C. rea(del(2)(p11)), D. der(5)t(5;11)(p11;?), E. del(6)(q16.1), F. i(17)(q10), G. der(18)t(7;18)(?;p11), H. der(19)t(19;22)(q13;?).


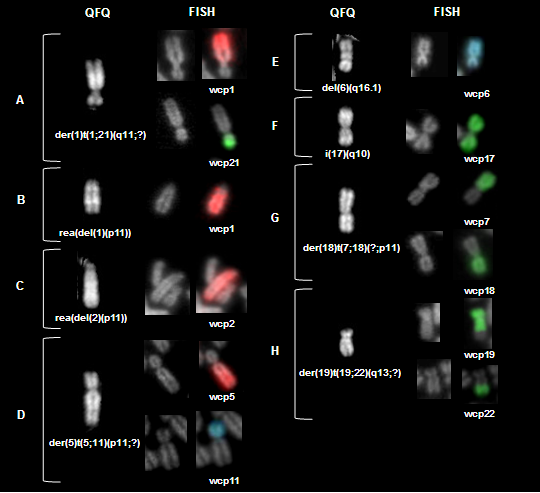

Supplement: Figure S3 — Panel of G166 chromosomal abnormalities identified through FISH analysis. (DOC) [file pone.0057462.s003.doc]
