# Supplementary material for: Delineating the Cytogenomic and Epigenomic Landscapes of Glioma Stem Cell Lines
Source: PLoS One. 2013 Feb 28;8(2):e57462. doi: 10.1371/journal.pone.0057462 (PMC3585345; doi:10.1371/journal.pone.0057462)

***Figure S4. Panel of GliNS2 chromosomal abnormalities identified through FISH analysis.*** Each aberration is described by means of QFQ-banded chromosomes and the corresponding FISH results. A. inv(del(1)(p34.3)), B.del(3)(p22?;p25?), C.der(2)t(2;20), D. der(5)t(2;5), E. del(10)(q21.3), F. der(6)t(3;6)(?;q27), G. der(21)t(6;21).


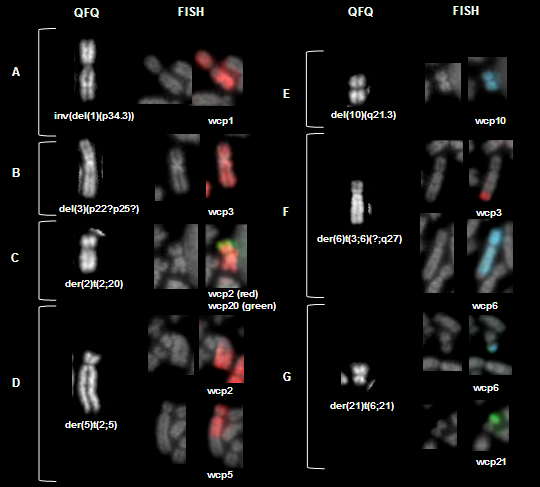

Supplement: Figure S4 — Panel of GliNS2 chromosomal abnormalities identified through FISH analysis. (DOC) [file pone.0057462.s004.doc]
