# Supplementary material for: Delineating the Cytogenomic and Epigenomic Landscapes of Glioma Stem Cell Lines
Source: PLoS One. 2013 Feb 28;8(2):e57462. doi: 10.1371/journal.pone.0057462 (PMC3585345; doi:10.1371/journal.pone.0057462)

***Figure S5. RT-PCR performed on RNA obtained from GBM2, G166, G179, GliSN2 and GBM7 cells.*** The figure shows the correlation between PTEN expression and genomic and epigenomic alterations. GBM2 and G166 cell lines showed no genomic alterations at PTEN locus. Anyway, differences in gene expression were appreciated. GBM2 cell line revealed no expression of PTEN gene, while G166 cell line displayed PTEN expression. This divergence is probably due to the differences in PTEN promoter methylation: GBM2 cell line shows methylation of PTEN promoter region and thus no PTEN expression, while G166 cell line had unmethylated PTEN promoter and consequently PTEN expression. GBM7 cell line showed a slight expression of PTEN gene, probably due to the mosaic alteration found at 10q23.3 locus. Also G179 and GliNS2 cell lines showed alterations in PTEN genomic locus (loss of 10q23.3) and these cell lines had no PTEN expression. HPRT housekeeping gene is used as endogenous control. n.d., not determined.


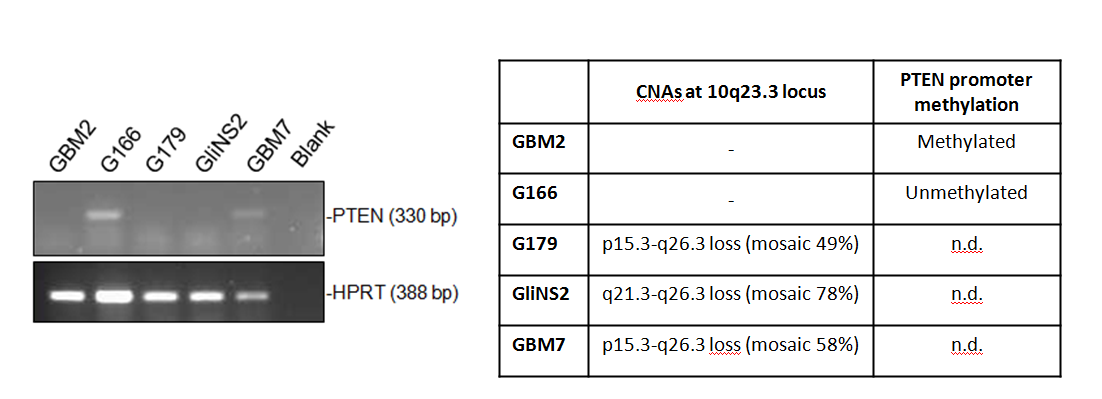

Supplement: Figure S5 — RT-PCR performed on RNA obtained from GBM2, G166, G179, GliSN2 and GBM7 cells. (DOC) [file pone.0057462.s005.doc]
