# Supplementary material for: Delineating the Cytogenomic and Epigenomic Landscapes of Glioma Stem Cell Lines
Source: PLoS One. 2013 Feb 28;8(2):e57462. doi: 10.1371/journal.pone.0057462 (PMC3585345; doi:10.1371/journal.pone.0057462)
Supplement: Figure S6 — Quantitative CpG methylation analysis of A) MGMT and B) PDGFB promoters. (DOC) [file pone.0057462.s006.doc]

***Figure S6. Quantitative CpG methylation analysis of A) MGMT and B) PDGFB promoters.***

Representative pyrograms showing the methylation profile of G144, G166 (GSCs) and CB660 (NSCs) cell lines. The potentially-methylated cytosines (C or T after bisulphite treatment) are highlighted in grey. Methylation levels at individual CpG positions are shown as the percentage of methylation above the respective positions. Sky-blue box = secure data; yellow box = doubtful data; red box = invalid data. Cut off values are based on the average methylation percentages of these specific CpGs in normal brain tissues and in whole blood.

**A) Cut off: x < 5% → unmethylated; 5% < x < 10% → intermediate methylation; x > 10% → methylated.**

Assay Name: MGMT

Sample ID: G144

Average CpG methylation: 88%

Assay Name: MGMT

Sample ID: G166

Average CpG methylation: 7%

Assay Name: MGMT

Sample ID: CB660

Average CpG methylation: 14%

**B) Cut off: x < 11% → unmethylated; x > 11% → methylated.**

Assay Name: PDGFB

Sample ID: G144

Average CpG methylation: 66%

Assay Name: PDGFB

Sample ID: G166

Average CpG methylation: 12%

Assay Name: PDGFB

Sample ID: CB660

Average CpG methylation: 7%
