# Supplementary material for: Delineating the Cytogenomic and Epigenomic Landscapes of Glioma Stem Cell Lines
Source: PLoS One. 2013 Feb 28;8(2):e57462. doi: 10.1371/journal.pone.0057462 (PMC3585345; doi:10.1371/journal.pone.0057462)
Supplement: Figure S7 — Real-time PCR data and promoter methylation of selected genes. (DOC) [file pone.0057462.s007.doc]

***Figure S7. Real-time PCR data and promoter methylation of selected genes.*** This analysis was performed in order to investigate the correlation between promoter methylation and gene expression. qRT-PCR assay on 2 genes with promoter CpG island hypomethylation in GBM2 cell line (MeDip-ChiIP data ) showed increased expression of the downstream gene (p<0.05). The chart shows values normalized relative to housekeeping gene (HPRT). Values are expressed as fold modulation relative to G144 cell line (methylated promoter). Data are the averages of two independent experiments.

**
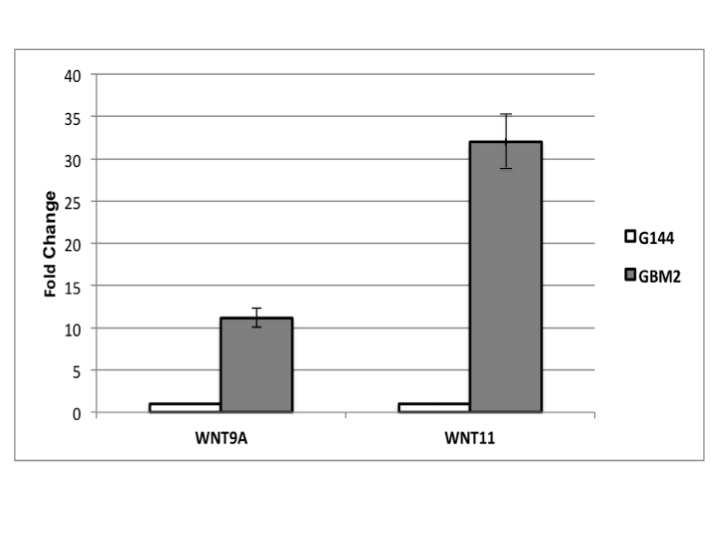
**
