# Supplementary material for: Delineating the Cytogenomic and Epigenomic Landscapes of Glioma Stem Cell Lines
Source: PLoS One. 2013 Feb 28;8(2):e57462. doi: 10.1371/journal.pone.0057462 (PMC3585345; doi:10.1371/journal.pone.0057462)
Supplement: Table S1 — List of STS markers. (DOC) [file pone.0057462.s008.doc]

***Table S1***. List of STS markers.

| **STS marker** | **Cytoband** | **Forward and reverse primers** | **PCR product size** |
| --- | --- | --- | --- |
| **D1S468** | 1p36.32 | AATTAACCGTTTTGGTCCT  GCGACACACACTTCCC | 173-191 bps |
| **D1S214** | 1p36.31 | CCGAATGACAAGGTGAGACT  AATGTTGTTTCCAAAGTGGC | 120-142 bps |
| **D1S508** | 1p36.23 | AGCTGGGGAATATATGTNTCATAT  TGTGGAAGGCCAACTC | 73-85 bps |
| **D1S228** | 1p36.21 | AACTGCAACATTGAAATGGC  GGGACCATAGTTCTTGGTGA | 116-129 bps |
| **D1S199** | 1p36.13 | GGTGACAGAGTGAGACCCTG  CAAAGACCATGTGCTCCGTA | 94-116 bps |
| **D1S2734** | 1p35.12 | GGTTCAAGGGATTCTCCTG  TGGCACTCAGACCTCAA | 108-134 bps |
| **D10S249** | 10p15.3 | AACTGGTTTTGGTAGTGAGA  GAGGTGCCCGCTAGTA | 118-134 bps |
| **D10S594** | 10p15.3 | GGGCAGCGTGCTGAGA  GCACCCAGATAGGCATAGAGA | 100-108 bps |
| **D10S558** | 10p15.3 | ATGAACATCACCAAGGCATATAG  ATAGTAGGCCGCCAGTCTC | 192-212 bps |
| **D10S552** | 10p15.1 | GTCCTTTAATCTGGGCTTTC  AATAGGTGGGGGCTTATG | 84-103 bps |
| **D10S1653** | 10p13 | CCTTTGGATAAAGCCTCCT  TATCATTGTCTCATCCGGG | 201-213 bps |
| **D10S197** | 10p12.1 | ACCACTGCACTTCAGGTGAC  GTGATACTGTCCTCAGGTCTCC | 161-173 bps |
| **D10S1686** | 10q23.1 | CTCTTCAGTTCCAACCACAC  ATAACACAGGGCCATTTAAG | 172-208 bps |
| **D10S185** | 10q23.33 | TCCTATGCTTTCATTTGCCA  CAAGACACACGATGTGCCAG | 143-159 bps |
| **D10S212** | 10q26.3 | GAAGTAAAGCAAGTTCTATCCACG  TCTGTGTACGTTGAAAATCCC | 189-201 bps |
| **D13S263** | 13q14.11 | CCTGGCCTGTTAGTTTTTATTGTTA  CCCAGTCTTGGGTATGTTTTTA | 145-165 bps |
| **D13S170** | 13q31.1 | TTGCACTGTGGAGATAAACACATAG  TCACATTGTCTTTTAAGGCAGGAG | 113-137 bps |
| **D13S159** | 13q32.2 | AGGCTGTGACTTTTAGGCCA  CCAGGCCACTTTTGATCTGT | 168-203 bps |
| **D13S285** | 13q34 | ATATATGCACATCCATCCATG  GGCCAAAGATAGATAGCAAGGTA | 92-106 bps |
