# Supplementary material for: Delineating the Cytogenomic and Epigenomic Landscapes of Glioma Stem Cell Lines
Source: PLoS One. 2013 Feb 28;8(2):e57462. doi: 10.1371/journal.pone.0057462 (PMC3585345; doi:10.1371/journal.pone.0057462)
Supplement: Table S2 — List of CNAs and mosaic level in GBM2 cell line. (DOC) [file pone.0057462.s009.doc]

***Table S2. List of CNAs and mosaic level in GBM2 cell line.*** *Abbreviations: Mb, megabases; CN, copy number; Amp, amplification; Null, nullisomy.*

| **Chromosome: nucleotides** | **Cytoband** | **Size (Mb)** | **log2ratio (CN)** | **Mosaic level (%)** |
| --- | --- | --- | --- | --- |
| 1: 40115450-40200491 | p34.2 | 0.09 | 0.43 (2.69) | Gain 69% |
| 1: 42392112-97117859 | p34.2-p21.3 | 54.73 | -0.54 (1.37) | Loss 63% |
| 1: 97252054-118688588 | p21.3-p12 | 21.44 | 0.68 (3.20) | Gain |
| 1: 202545868-202928323 | q32.1 | 0.38 | 0.72 (3.29) | Gain |
| 1: 247071254-247179432 | q44 | 0.11 | -0.52 (1.39) | Loss 61% |
| 2: 28993-7075715 | p25.3-p25.1 | 7.05 | -0.56 (1.36) | Loss 64% |
| 2: 15994227-16495933 | p24.3 | 0.50 | 3.49 (22.47) | Amp |
| 2: 74968621-84778404 | p13.1-p11.2 | 9.81 | -0.51 (1.40) | Loss 60% |
| 2: 102162243-102799123 | q12.1 | 0.64 | -0.62 (1.30) | Loss 70% |
| 2: 119960366-120154667 | q14.2 | 0.19 | 3.77 (27.28) | Amp |
| 2: 137097438-148401386 | q22.1-q23.1 | 11.30 | -0.50 (1.41) | Loss 59% |
| 3: 261100-2117482 | p26.3 | 1.85 | -0.74 (1.20) | Loss 80% |
| 3: 53506299-56305273 | p21.1-p14.3 | 2.80 | -0.36 (1.56) | Loss 44% |
| 3: 58527174-90264318 | p14.2-p11.1 | 31.74 | -0.42 (1.49) | Loss 51% |
| 3: 97182255-99151688 | q11.2 | 1.97 | -0.63 (1.29) | Loss 71% |
| 4: 504250-191121240 | p16.3-q35.2 | 190.62 | -0.36 (1.56) | Loss 44% |
| 4: 42321145-46083064 | p13-p12 | 3.76 | -0.64 (1.28) | Loss 72% |
| 4: 53755407-55454032 | q12 | 1.70 | 3.70 (25.99) | Amp |
| 4: 96640205-97766572 | q22.3 | 1.13 | -0.91 (1.06) | Loss 94% |
| 5: 16504399-17344637 | p15.1 | 0.84 | 0.39 (2.62) | Gain 62% |
| 5: 31448403-32633036 | p13.3 | 1.19 | 0.36 (2.57) | Gain 57% |
| 5: 50714979-50725758 | q11.2 | 0.01 | -0.71 (1.22) | Loss 78% |
| 5: 68434443-68900170 | q13.2 | 0.47 | 0.45 (2.73) | Gain 73% |
| 5: 133685191-134218892 | q31.1 | 0.53 | 0.39 (2.62) | Gain 62% |
| 5: 137520257-137736207 | q31.2 | 0.22 | 0.47 (2.77) | Gain 77% |
| 6: 45622847-52239790 | p12.3-p12.1 | 6.62 | -0.43 (1.48) | Loss 52% |
| 6: 107207720-108875190 | q21 | 1.67 | 0.40 (2.64) | Gain 64% |
| 7: 797178-158568562 | p22.3-q36.3 | 157.77 | 0.34 (2.53) | Gain 53% |
| 7: 4805154-6811413 | p22.1 | 2.01 | 0.72 (3.29) | Gain |
| 7: 23171848-23634478 | p15.3 | 0.46 | 0.67 (3.18) | Gain |
| 7: 44539142-45112595 | p13 | 0.57 | 0.67 (3.18) | Gain |
| 7: 62470818-77988860 | q11.21-q21.11 | 15.52 | 0.60 (3.03) | Gain |
| 7: 97718447-101884346 | q21.3-q22.1 | 4.17 | 0.60 (3.03) | Gain |
| 7: 137442322-140266845 | q34 | 2.83 | 0.45 (2.73) | Gain 73% |
| 7: 148174347-148879836 | q36.1 | 0.71 | 0.70 (3.25) | Gain |
| 7: 151285611-152089322 | q36.1 | 0.80 | 0.74 (3.34) | Gain |
| 8: 62364458-146250824 | q11.21-q24.3 | 83.89 | -1.05 (0.97) | Loss |
| 8: 139224333-146250824 | q24.23-q24.3 | 7.03 | -0.33 (1.59) | Loss 41% |
| 9: 21399600-26790967 | p21.3-p21.2 | 5.39 | -1.18 (0.88) | Loss |
| 9: 21795070-21999182 | p21.3 | 0.20 | -2.80 (0.29) | Null |
| 10: 1221842-5031869 | p15.3-p15.1 | 3.81 | -0.47 (1.44) | Loss 56% |
| 10: 43673512-44424478 | q11.21 | 0.75 | -0.80 (1.15) | Loss 85% |
| 11: 186766-47245293 | p15.5-p11.2 | 47.06 | -0.68 (1.25) | Loss 75% |
| 11: 55572629-133220697 | q11-q25 | 77.65 | -0.37 (1.55) | Loss 45% |
| 11: 47276165-47417895 | p11.2 | 0.14 | 0.44 (2.71) | Gain 71% |
| 11: 48038103-55460512 | p11.2-q11 | 7.42 | -0.67 (1.23) | Loss 77% |
| 11: 55572629-61209726 | q11-q12.2 | 5.64 | -0.68 (1.25) | Loss 75% |
| 12: 10256380-19351142 | p13.2-p12.3 | 9.10 | -0.81 (1.14) | Loss 86% |
| 12: 31433413-32930828 | p11.21 | 1.50 | 0.40 (2.64) | Gain 64% |
| 12: 47809770-48281737 | q13.12 | 0.47 | 0.54 (2.91) | Gain 91% |
| 12: 51837505-52164846 | q13.13 | 0.33 | 0.44 (2.71) | Gain 71% |
| 13: 18601503-114077263 | q12.11-q34 | 95.48 | -0.81 (1.14) | Loss 86% |
| 14: 72852631-72989548 | q24.2-q24.3 | 0.14 | 0.76 (3.39) | Gain |
| 16: 5995867-8440586 | p13.3-p13.2 | 2.45 | -0.50 (1.41) | Loss 59% |
| 16: 57847060-64965376 | q21 | 7.12 | -0.49 (1.42) | Loss 58% |
| 16: 74907521-79361611 | q23.1-q23.2 | 4.45 | -0.45 (1.46) | Loss 54% |
| 17: 6185227-9990093 | p13.2-p13.1 | 3.81 | -0.54 (1.38) | Loss 62% |
| 17: 28281765-29607623 | q11.2-q12 | 1.33 | -0.49 (1.42) | Loss 58% |
| 18: 895893-6827570 | p11.32-p11.31 | 5.93 | -0.50 (1.41) | Loss 59% |
| 18: 23784726-76083258 | q12.1-q23 | 52.30 | -0.50 (1.41) | Loss 59% |
| 18: 33105541-41038319 | q12.2-q12.3 | 7.93 | -0.71 (1.22) | Loss 78% |
| 19: 37424589-41195674 | q13.11-q13.12 | 3.77 | 0.72 (3.29) | Gain |
| 19: 41658326-63784527 | q13.12-q13.43 | 22.13 | -0.41 (1.51) | Loss 49% |
| 19: 63539457-63784527 | q13.43 | 0.25 | -0.72 (1.21) | Loss 79% |
| 20: 18380-26023984 | p13-p11.1 | 26.01 | -0.31 (1.61) | Loss 39% |
| X: 7279021-7837167 | p22.31 | 0.56 | -0.61 (1.31) | Loss 69% |
| Y: 2783545-23283889 | p11.31-q11.223 | 20.50 | -0.85 (1.11) | Loss 89% |
| Y: 8366080-10144057 | p11.2 | 1.78 | -1.46 (0.73) | Loss |
| Y: 20337999-23283889 | q11.222-q11.223 | 2.95 | -1.14 (0.91) | Loss |
