# Supplementary material for: Delineating the Cytogenomic and Epigenomic Landscapes of Glioma Stem Cell Lines
Source: PLoS One. 2013 Feb 28;8(2):e57462. doi: 10.1371/journal.pone.0057462 (PMC3585345; doi:10.1371/journal.pone.0057462)
Supplement: Table S3 — List of CNAs and mosaic level in GBM7 cell line. (DOC) [file pone.0057462.s010.doc]

***Table S3. List of CNAs and mosaic level in GBM7 cell line.*** Abbreviations: Mb, megabases; CN, copy number; Amp, amplification; Null, nullisomy.

| **Chromosome: nucleotides** | **Cytoband** | **Size (Mb)** | **log2ratio (CN)** | **Mosaic level (%)** |
| --- | --- | --- | --- | --- |
| 1: 6080947-9801999 | p36.31-p36.22 | 3.72 | -0.52 (1.39) | Loss 61% |
| 1: 13739644-13997705 | p36.21 | 0.03 | 3.99 (31.78) | Amp |
| 1: 14175589-17266375 | p36.21-p36.13 | 3.09 | -0.55 (1.37) | Loss 63% |
| 1: 17313918-17956435 | p36.13 | 0.64 | 4.09 (34.06) | Amp |
| 1: 20316451-20548367 | p36.12 | 0.23 | -0.61 (1.31) | Loss 69% |
| 1: 22334103-24329247 | p36.12-p36.11 | 1.99 | -0.49 (1.42) | Loss 58% |
| 1: 25256867-25446281 | p36.11 | 0.19 | 3.93 (30.48) | Amp |
| 1: 32347943-38364276 | p35.1-p34.3 | 6.02 | -0.58 (1.34) | Loss 66% |
| 1: 33562581-34436532 | p35.1-p34.3 | 0.87 | -1.08 (0.95) | Loss |
| 1: 36835076-37658465 | p34.3 | 0.82 | -1.14 (0.91) | Loss |
| 1: 38804045-39671515 | p34.3-p34.2 | 0.87 | 3.80 (27.86) | Amp |
| 1: 39739948-40700874 | p34.2 | 0.96 | -0.51 (1.40) | Loss 60% |
| 1: 228316266-228439117 | q42.13 | 0.12 | -0.57 (1.35) | Loss 65% |
| 2: 25607622-26386895 | p22.3 | 0.78 | 0.65 (3.12) | Gain |
| 2: 32038185-32812604 | p22.3 | 0.77 | 0.70 (3.25) | Gain |
| 2: 85405164-85641567 | p11.2 | 0.24 | 0.68 (3.20) | Gain |
| 5: 16642984-17344637 | p15.1 | 0.70 | 0.54 (2.91) | Gain 91% |
| 5: 68203407-68768327 | q13.1-q13.2 | 0.57 | 0.62 (3.07) | Gain |
| 5:133685191-134215013 | q31.1 | 0.53 | 0.53 (2.89) | Gain 89% |
| 5: 137520257-137736207 | q31.2 | 0.22 | 0.70 (3.25) | Gain |
| 6: 63034990-170732174 | q11.1-q27 | 107.70 | -0.39 (1.53) | Loss 47% |
| 6: 117308229-123038149 | q22.2-q22.31 | 5.73 | -1.58 (0.67) | Loss |
| 7: 797178-158568562 | p22.3-q36.3 | 157.80 | 0.55 (2.93) | Gain 93% |
| 7: 4298390-6811413 | p22.2-p22.1 | 2.51 | 0.96 (3.89) | Gain |
| 7: 23171848-23537259 | p15.3 | 0.37 | 0.96 (3.89) | Gain |
| 7: 55680513-56754495 | p11.2 | 1.07 | 0.96 (3.89) | Gain |
| 7: 62153388-75446606 | q11.21-q11.23 | 13.29 | 0.92 (3.78) | Gain |
| 7: 97718447-102735489 | q21.3-q22.1 | 5.02 | 0.82 (3.53) | Gain |
| 7: 137295729-140266986 | q33-q34 | 2.97 | 0.85 (3.63) | Gain |
| 7: 151379586-152089322 | q36.1 | 0.71 | 1.01 (4.03) | Gain |
| 8: 103637144-104171398 | q22.3 | 0.53 | 0.69 (3.23) | Gain |
| 9: 6883176-10430855 | p24.1-p23 | 3.55 | -0.51 (1.40) | Loss 60% |
| 9: 17185565-18940918 | p22.2-p21.3 | 1.76 | -1.01 (0.99) | Loss |
| 9: 21231271-22889784 | p21.3 | 1.66 | -2.69 (0.31) | Null |
| 9: 22992118-26455189 | p21.3-p21.1 | 3.46 | -0.65 (1.27) | Loss 73% |
| 9: 70533868-134968633 | q21.11-q34.2 | 64.44 | 0.31 (2.48) | Gain 48% |
| 9: 73530120-74352540 | q21.13 | 0.82 | 0.70 (3.25) | Gain |
| 9: 103237866-103489305 | q31.1 | 0.25 | -0.57 (1.35) | Loss 65% |
| 9: 113511997-115173153 | q31.3-q32 | 1.66 | 0.61 (3.05) | Gain |
| 9: 139498321-139853505 | q34.3 | 0.36 | 0.48 (2.79) | Gain 79% |
| 10: 138006-135254661 | p15.3-q26.3 | 135.12 | -0.49 (1.42) | Loss 58% |
| 10: 138006-5841071 | p15.3-p15.1 | 5.70 | -0.69 (1.24) | Loss 76% |
| 10: 43804087-63620111 | q11.21-q21.2 | 19.82 | -0.75 (1.19) | Loss 81% |
| 10: 72131576-73246511 | q22.1 | 1.12 | -0.70 (1.23) | Loss 77% |
| 12: 31570386-32798334 | p11.21 | 1.23 | 0.47 (2.77) | Gain 77% |
| 12: 48794402-49961914 | q13.13 | 1.16 | 0.55 (2.93) | Gain 93% |
| 12: 55113747-55366355 | q13.2-q13.3 | 0.25 | 0.71 (3.27) | Gain |
| 12: 108799898-109689665 | q24.11-q24.13 | 0.89 | 0.54 (2.91) | Gain 91% |
| 12: 118603529-123038369 | q24.23-q24.31 | 4.44 | 0.41 (2.66) | Gain 66% |
| 14: 34106419-35080562 | q13.2 | 0.97 | 0.47 (2.77) | Gain 77% |
| 14: 49144127-50000702 | q22.1 | 0.86 | 0.45 (2.73) | Gain 73% |
| 14: 101414850-102568451 | q32.31-q32.33 | 1.15 | 0.39 (2.62) | Gain 62% |
| 15: 62165578-62723076 | q22.31 | 0.56 | 0.55 (2.93) | Gain 93% |
| 16: 298656-479287 | p13.3 | 0.18 | 0.59 (3.01) | Gain |
| 16: 67217316-68044948 | q21-q23.1 | 0.83 | 0.54 (2.91) | Gain 91% |
| 16: 88283847-88504677 | q24.3 | 0.22 | 0.69 (3.23) | Gain |
| 17: 202609-1287282 | p13.3 | 1.08 | 0.54 (2.91) | 91% |
| 17: 70548472-70977154 | q25.1 | 0.43 | 0.60 (3.03) | Gain |
| 19: 231880-63592894 | p13.3-q13.43 | 63.36 | 0.60 (3.03) | Gain |
| 19: 40990735-43085611 | q13.12-q13.13 | 2.09 | 0.82 (3.53) | Gain |
| 20: 18380-62266665 | p13-q13.33 | 62.25 | 0.54 (2.91) | Gain 91% |
| 20: 3037684-6044872 | p13-p12.3 | 30.07 | 0.71 (3.27) | Gain |
| 20: 32125088-35275770 | q11.22-q11.23 | 31.51 | 0.87 (3.66) | Gain |
| 22: 15979229-16597551 | q11.1-q11.21 | 0.62 | 0.59 (3.01) | Gain |
| X: 23836039-24138277 | p22.11 | 0.30 | 0.61 (3.05) | Gain |
| X: 124892325-125511604 | q25 | 0.62 | 0.70 (3.25) | Gain |
| Y: 4411696-23283889 | p11.2-q11.223 | 18.87 | -0.77 (1.17) | Loss 83% |
