# Supplementary material for: Delineating the Cytogenomic and Epigenomic Landscapes of Glioma Stem Cell Lines
Source: PLoS One. 2013 Feb 28;8(2):e57462. doi: 10.1371/journal.pone.0057462 (PMC3585345; doi:10.1371/journal.pone.0057462)
Supplement: Table S4 — List of CNAs and mosaic level in G166 cell line. (DOC) [file pone.0057462.s011.doc]

***Table S4. List of CNAs and mosaic level in G166 cell line.*** Abbreviations: Mb, megabases; CN, copy number; Amp, amplification; Null, nullisomy.

| **Chromosome: nucleotides** | **Cytoband** | **Size (Mb)** | **log2ratio (CN)** | **Mosaic level (%)** |
| --- | --- | --- | --- | --- |
| 1: 554068-120449334 | p36.33-p12 | 119.90 | 0.53 (2.89) | Gain 89% |
| 1: 143638935-20925978 | q21.1-q32.2 | 65.62 | 0.53 (2.89) | Gain 89% |
| 1: 210452628-210866896 | q32.3 | 414.27 | 0.52 (2.87) | Gain 87% |
| 1: 231008707-247179432 | q42.2-q44 | 16.17 | 0.50 (2.83) | Gain 83% |
| 2: 94999487-223786365 | q11.1-q36.1 | 128.79 | 0.46 (2.75) | Gain 75% |
| 2: 231629072-231908002 | q37.1 | 278.90 | -0.34 (1.58) | Loss 42% |
| 3: 224527-199288361  3:197927914-199288361 | p26.3-q29  q29 | 199.07  1.36 | 0.55 (2.93)  0.91 (3.76) | Gain 93%  Gain |
| 4: 62247-35612519 | p16.3-p14 | 35.55 | 0.53 (2.89) | Gain 89% |
| 5: 14731486-16207795 | p15.2-p15.1 | 1.48 | -0.82 (1.13) | Loss 87% |
| 5: 65035668-180617248 | q12.3-q35.3 | 115.58 | 0.55 (2.93) | Gain 93% |
| 6: 352263-96345686 | p25.3-q16.1 | 95.99 | 0.59 (3.01) | Gain |
| 6: 55221329-57298946 | p12.1-p11.2 | 2.08 | 0.89 (3.71) | Gain |
| 6: 97170758-97479498 | q16.1 | 0.31 | 0.52 (2.87) | Gain 87% |
| 7: 149068-69538849 | p22.3-q11.2 | 69.39 | 0.46 (2.75) | Gain 75% |
| 7: 130846465-134844529 | q32.3-q33 | 4.00 | 0.56 (2.95) | Gain 95% |
| 8: 104266942-111730488 | q22.3-q23.2 | 7.46 | -0.81 (1.14) | Loss 86% |
| 9: 92949868-140128884 | q22.2-q34.3 | 47.18 | 0.51 (2.85) | Gain 85% |
| 10: 21854263-21980801 | p12.31 | 0.13 | 0.87 (3.66) | Gain |
| 10: 76884518-83652747 | q22.2-q23.1 | 6.77 | 0.61 (3.05) | Gain |
| 10: 93691959-94383592 | q23.32-q23.33 | 0.69 | 0.49 (2.81) | Gain 81% |
| 11: 186766-60172966  11: 34482220-36493813 | p15.5-q12.2  p13-p12 | 59.99  2.01 | 0.50 (2.83)  0.82 (3.53) | Gain 83%  Gain |
| 11: 78137964-79025590 | q14.1 | 0.89 | 0.31 (2.48) | Gain 48% |
| 11: 112268205-112447353 | q23.1 | 0.18 | 0.69 (3.23) | Gain |
| 12: 89969071-90084523 | q21.33 | 0.12 | 0.42 (2.68) | Gain 68% |
| 14: 19508645-23619976 | q11.2-q12 | 4.11 | 0.52 (2.87) | Gain 87% |
| 14: 67925202-68324499 | q24.1 | 0.40 | 0.97 (3.92) | Gain |
| 16: 15441429-15867785 | p13.11 | 0.43 | 0.50 (2.83) | Gain 83% |
| 17: 21938906-78623371  17: 29622049-30253211 | p11.2-q25.3  q12 | 56.68  0.63 | 0.51 (2.85)  0.87 (3.66) | Gain 85%  Gain |
| 17: 43333046-46127533 | q21.32-q21.33 | 2.79 | 1.12 (4.35) | Amp |
| 17: 75294309-75606495 | q25.3 | 0.31 | 1.28 (4.86) | Amp |
| 18: 170029-12149645 | p11.32-p11.21 | 11.98 | -0.86 (1.10) | Loss 90% |
| 19: 231880-1443256 | p13.3 | 1.21 | 0.51 (2.85) | Gain 85% |
| 19: 18531065-63784527  19: 33277715-37126981 | p13.11-q13.43  q12-q13.11 | 45.25  3.85 | 0.45 (2.73)  0.94 (3.84) | Gain 73%  Gain |
| 20: 18380-62363774  20: 42724115-43087198 | p13-q13.33  q13.12 | 62.35  0.36 | 0.89 (3.71)  1.40 (5.28) | Gain  Amp |
| 21: 13548935-15109123 | q11.2 | 1.56 | 0.51 (2.85) | Gain 85% |
| 22: 41106142-41453794 | q13.2 | 0.35 | 0.78 (3.43) | Gain |
| X: 2710116-154494790 | p22.33-q28 | 151.79 | 1.63 (6.19) | Amp |
| Y: 2907850-6493634 | p11.31-p11.22 | 3.59 | 0.43 (2.69) | Gain 69% |
| Y: 6652668-6993728 | p11.2 | 0.34 | -1.82 (0.57) | Loss |
| Y: 7740091-10144057 | p11.2 | 2.40 | -1.92 (0.53) | Loss |
| Y: 13254511-13432175 | q11.21 | 0.18 | -2.63 (0.32) | Null |
| Y: 13852177-14356269 | q11.21-q11.221 | 0.50 | -1.12 (0.92) | Loss |
| Y: 15388603-23283748 | q11.221-q11.223 | 7.90 | -1.51 (0.70) | Loss |
