# Supplementary material for: Delineating the Cytogenomic and Epigenomic Landscapes of Glioma Stem Cell Lines
Source: PLoS One. 2013 Feb 28;8(2):e57462. doi: 10.1371/journal.pone.0057462 (PMC3585345; doi:10.1371/journal.pone.0057462)
Supplement: Table S5 — List of CNAs and mosaic level in G179 cell line. (DOC) [file pone.0057462.s012.doc]

***Table S5. List of CNAs and mosaic level in G179 cell line.*** *Abbreviations: Mb, megabases; CN, copy number; Amp, amplification; Null, nullisomy.*

| **Chromosome: nucleotides** | **Cytoband** | **Size (Mb)** | **log2ratio (CN)** | **Mosaic level (%)** |
| --- | --- | --- | --- | --- |
| 1: 1009416-117970468  1: 6171802-47616814  1:57187611-117968849 | p36.33-p12  p36.31-p33  p32.2-p12 | 116.96  41.45  60.78 | -0.36 (1.56)  -0.44 (1.47)  -0.27 (1.66) | Loss 44%  Loss 53%  Loss 34% |
| 2: 2757409-242464793  2: 96804902-96911470 | p25.3-q37.3  q11.2 | 239.71  0.11 | -0.21 (1.73)  -0.58 (1.34) | Loss 27%  Loss 66% |
| 3: 95088005-199288361  3: 153319548-160029671 | q11.2-q29  q25.1-q25.33 | 104.20  6.71 | 0.25 (2.38)  -0.43 (1.48) | Gain 38%  Loss 52% |
| 4: 296641-48758199 | p16.3-p11 | 48.46 | -0.17 (1.78) | Loss 22% |
| 4: 190887201-191121344 | q35.2 | 0.23 | -0.43 (1.48) | Loss 52% |
| 5: 327794-180617248 | p15.33-q35.3 | 180.29 | -0.45 (1.46) | Loss 54% |
| 6: 352263-170734368  6: 53794832-56672592 | p25.3-q27  p12.1 | 170.38  2.88 | 0.24 (2.36)  0.41 (2.66) | Gain 36%  Gain 66% |
| 7: 149068-57327581 | p22.3-p11.2 | 57.18 | 0.76 (3.39) | Gain |
| 7: 62153388-158781538  7:63623952-75982831  7: 77260971-96600328  7: 106623543-127029210 | q11.21-q36.3  q11.21-q11.23  q11.23-q21.3  q22.3-q32.1 | 96.63  12.36  19.34  20.41 | 0.42 (2.68)  0.25 (2.38)  0.53 (2.89)  0.53 (2.89) | Gain 68%  Gain 38%  Gain 89%  Gain 89% |
| 8: 181330-146250965  8: 143990549-145713213 | p23.3-q24.3  q24.3 | 146.07  1.72 | 0.32 (2.50)  0.16 (2.23) | Gain 50%  Gain 23% |
| 9: 204167-31181738  9: 21231271-22136826 | p24.3-p21.1  p21.3 | 30.98  0.91 | -0.62 (1.30)  -4.23 (0.11) | Loss 70%  Null |
| 10: 138006-135222624 | p15.3-q26.3 | 135.08 | -0.41 (1.51) | Loss 49% |
| 11: 274638-830396 | p15.5 | 0.56 | -0.21 (1.73) | Loss 27% |
| 11: 46194612-49187069 | p11.2-p11.12 | 2.99 | -0.17 (1.78) | Loss 22% |
| 12: 11678112-44611808 | p13.2-q13.11 | 32.92 | -0.58 (1.34) | Loss 66% |
| 13: 18601503-113373947  13: 18601503-50679198 | q12.11-q34  q12.11-q14.3 | 94.77  32.08 | -0.22 (1.72)  -0.43 (1.48) | Loss 28%  Loss 52% |
| 14: 72432103-75067922 | q24.2-q24.3 | 2.64 | -0.25 (1.68) | Loss 32% |
| 15: 20335687-100168859 | q11.2-q26.3 | 79.83 | -0.56 (1.36) | Loss 64% |
| 16: 36566-88638909 | p13.3-q24.3 | 88.60 | 0.26 (2.39) | Gain 39% |
| 17: 38832282-78623371  17: 47062751-52081236 | q21.31-q25.3  q21.33-q22 | 39.79  5.02 | 0.28 (2.43)  0.43 (2.69) | Gain 43%  Gain 69% |
| 19: 34522344-63784322 | q12-q13.43 | 29.26 | -0.65 (1.27) | Loss 73% |
| 20: 61633659-62135057 | q21.3 | 0.50 | -0.25 (1.68) | Loss 32% |
| 21: 27093082-27443730 | q21.3 | 0.35 | -0.86 (1.10) | Loss 90% |
| 21: 41367553-42098261 | q22.2-q22.3 | 0.73 | -0.91 (1.06) | Loss 94% |
| X: 2710116-52710832 | p22.33-p11.22 | 50.00 | -0.38 (1.54) | Loss 46% |
| X: 52857751-154494790  X:56279086-56609862 | p11.22-q28  p11.21-p11.1 | 101.64  0.33 | 0.48 (2.79)  1.34 (5.06) | Gain 79%  Amp |
| Y: 2783545-5531670 | p11.31-p11.2 | 2.75 | -0.37 (1.55) | Loss 45% |
| Y: 6652668-10144057 | p11.2 | 3.50 | -0.84 (1.12) | Loss 88% |
| Y: 12550190-57432638 | q11.21-q11.223 | 44.88 | -0.80 (1.15) | Loss 85% |
