# Supplementary material for: Delineating the Cytogenomic and Epigenomic Landscapes of Glioma Stem Cell Lines
Source: PLoS One. 2013 Feb 28;8(2):e57462. doi: 10.1371/journal.pone.0057462 (PMC3585345; doi:10.1371/journal.pone.0057462)
Supplement: Table S6 — List of CNAs and mosaic level in GliNS2 cell line. (DOC) [file pone.0057462.s013.doc]

***Table S6. List of CNAs and mosaic level in GliNS2 cell line.*** Abbreviations: Mb, megabases; CN, copy number; Amp, amplification; Null, nullisomy.

| **Chromosome: nucleotides** | **Cytoband** | **Size (Mb)** | **log2ratio (CN)** | **Mosaic level (%)** |
| --- | --- | --- | --- | --- |
| 1: 1039674-39176138 | p36.33-p34.3 | 38.14 | -0.84 (1.12) | Loss 88% |
| 1: 39304203-119857728  1: 109624408-1096805610 | p34.3-p12  p13.3 | 80.55  0.06 | -0.28 (1.65)  0.38 (2.60) | Loss 35%  Gain 40% |
| 1: 246547373-247179291 | q44 | 0.63 | 0.40 (2.64) | Gain 64% |
| 2: 28644657-28717001 | p23.2 | 0.07 | 0.66 (3.16) | Gain |
| 2: 86106863-86180963 | p11.2 | 0.07 | -0.85 (1.11) | Loss 89% |
| 2: 208980214-216441453 | q33.3-q35 | 7.46 | -0.90 (1.07) | Loss 93% |
| 3: 52521925-52557261 | p21.1 | 0.04 | 0.59 (3.01) | Gain |
| 3: 57079217-57174834 | p14.3 | 0.10 | 0.72 (3.29) | Gain |
| 4: 9708394-10292183 | p16.1 | 0.58 | 0.50 (2.83) | Gain 83% |
| 4: 35769767-36791394 | p14 | 1.02 | -0.69 (1.24) | Loss 76% |
| 4: 65558477-66268186 | q13.1 | 0.71 | -0.87 (1.09) | Loss 91% |
| 5: 26982169-29938908 | p14.1-p13.3 | 2.96 | -1.01 (0.99) | Loss |
| 6: 204528-295265 | p25.3 | 0.09 | -0.57 (1.35) | Loss 65% |
| 6: 352263-3687477 | p25.3-p25.2 | 3.34 | 0.45 (2.73) | Gain 73% |
| 6: 7126373-11134690 | p24.3-p24.3 | 4.01 | -0.74 (1.20) | Loss 80% |
| 6: 20542402-22466783 | p22.3 | 1.92 | -0.94 (1.04) | Loss 96% |
| 6: 24410015-26379149 | p22.2-p22.1 | 1.97 | -0.78 (1.16) | Loss 84% |
| 6: 45987212-46088378 | p12.3 | 0.10 | -0.66 (1.27) | Loss 73% |
| 6: 46400848-46869563 | p12.3 | 0.47 | -0.87 (1.09) | Loss 91% |
| 6: 112486048-112681877 | q21 | 0.20 | 0.68 (3.20) | Gain |
| 6: 149818985-170700202 | q25.1-q27 | 20.88 | -0.82 (1.13) | Loss 87% |
| 7: 149068-158781538 | p11.21-q36.3 | 158.63 | 0.54 (2.91) | Gain 91% |
| 9: 21733210-29592035  9: 21980322-21999182 | p21.3-p21.1  p21.3 | 7.86  0.02 | -0.90 (1.07)  -3.93 (0.13) | Loss 93%  Null |
| 10: 14909740-15020635 | p13 | 0.11 | -1.06 (0.96) | Loss |
| 10: 70888043-135254513  10: 89790867-91085657 | q21.3-q26.3  q23.31 | 64.37  1.30 | -0.71 (1.22)  -3.07 (0.24) | 78%  Null |
| 11: 36359473-36613802 | p13-p12 | 0.25 | 0.67 (3.18) | Gain |
| 11: 130772681-132926137 | q25 | 2.15 | 0.34 (2.53) | Gain 53% |
| 12: 499967-239453 | p13.33 | 0.19 | 0.39 (2.62) | Gain 62% |
| 12: 52635534-56517093 | q13.33-q14.1 | 3.88 | -0.83 (1.13) | Loss 87% |
| 12: 119121736-119272256 | q24.31 | 0.15 | 0.50 (2.83) | Gain 83% |
| 14:20767432-36815493 | q11.2-q13.3 | 16.05 | -0.83 (1.13) | Loss 87% |
| 15: 19109124-20425188 | q11.2 | 1.32 | -0.38 (1.54) | Loss 46% |
| 16: 4736124-4793985 | p13.3 | 0.06 | 0.63 (3.10) | Gain |
| 17: 7401333-7448289 | p13.1 | 0.05 | 0.56 (2.95) | Gain 95% |
| 17: 17757522-17910300 | p11.2 | 0.15 | 0.56 (2.95) | Gain 95% |
| 17: 35384599-35478201 | q12-q21.1 | 0.09 | 0.52 (2.87) | Gain 87% |
| 18: 5226658-12149645 | p11.31-p11.21 | 6.92 | -0.88 (1.09) | Loss 91% |
| 18: 66627672-74128538 | q22.2-q23 | 7.50 | -0.71 (1.22) | Loss 78% |
| 19: 50088113-50207669 | q13.32 | 0.12 | 0.40 (2.64) | Gain 64% |
| 20: 39462451-39555803 | q12 | 0.09 | 0.74 (3.34) | Gain |
| 20: 45308362-45473055 | q13.12 | 0.16 | 0.51 (2.85) | Gain 85% |
| 22: 26607803-26829059 | q12.1 | 0.22 | 0.43 (2.69) | Gain 69% |
| 22: 27494498-27910945 | 12.1-q12.2 | 0.42 | 0.42 (2.68) | Gain 68% |
