# Supplementary material for: Delineating the Cytogenomic and Epigenomic Landscapes of Glioma Stem Cell Lines
Source: PLoS One. 2013 Feb 28;8(2):e57462. doi: 10.1371/journal.pone.0057462 (PMC3585345; doi:10.1371/journal.pone.0057462)
Supplement: Table S7 — Percentages of methylated and unmethylated CGIs in GSCs, foetal NSCs, PBL pool and GBM FFPE tissues, classified in the different functional genomic regions. (DOC) [file pone.0057462.s014.doc]

***Table S7. Percentages of methylated and unmethylated CGIs in GSCs, foetal NSCs, PBL pool and GBM FFPE tissues***, classified in the different functional genomic regions.

| **Cell line** | **% of unmethylated CGIs** | | | | | **% of methylated CGIs** | | | | |
| --- | --- | --- | --- | --- | --- | --- | --- | --- | --- | --- |
|  | **Promoter** | **Inside** | **Downstream** | **Divergent Promoter** | **Unknown** | **Promoter** | **Inside** | **Downstream** | **Divergent Promoter** | **Unknown** |
| **GBM2** | 19.9 | 30.7 | 2.0 | 1.7 | 4.9 | 7.0 | 24.7 | 3.0 | 0.6 | 5.5 |
| **G144** | 17.2 | 29.7 | 2.0 | 1.7 | 4.1 | 9.8 | 25.7 | 3.0 | 0.6 | 6.2 |
| **G166** | 22.2 | 39.5 | 3.0 | 1.9 | 6.0 | 5.0 | 15.7 | 2.0 | 0.4 | 4.3 |
| **CB660SP** | 12.7 | 20.2 | 1.9 | 1.2 | 2.9 | 14.4 | 36.3 | 3.3 | 0.9 | 6.2 |
| **CB660** | 23.0 | 33.6 | 2.8 | 1.9 | 4.4 | 4.4 | 22.7 | 2.4 | 0.3 | 4.5 |
| **PBL pool** | 24.2 | 38.9 | 3.4 | 1.9 | 5.3 | 3.4 | 16.2 | 1.5 | 0.4 | 4.8 |
| **GBM FFPE tissues** | 14.9 | 28.2 | 2.2 | 1.3 | 3.8 | 12 | 29 | 2.9 | 1 | 4.7 |
