# Supplementary material for: Delineating the Cytogenomic and Epigenomic Landscapes of Glioma Stem Cell Lines
Source: PLoS One. 2013 Feb 28;8(2):e57462. doi: 10.1371/journal.pone.0057462 (PMC3585345; doi:10.1371/journal.pone.0057462)
Supplement: Table S8 — Methylation status gene promoters associated to GBM pathogenesis. (DOC) [file pone.0057462.s015.doc]

***Table S8. Methylation status gene promoters associated to GBM pathogenesis.* Red, methylated gene promoter; green, unmethylated gene promoter; n.d., not determined**

| **Gene** | **Cytoband** | **GBM2** | **G144** | **G166** | **GBM FFPE** |
| --- | --- | --- | --- | --- | --- |
| **RASSF1A** | 3p21.3 | n.d. |  |  |  |
| **CDKN2A** | 9p21 |  |  |  |  |
| **MGMT** | 10q26 |  |  |  |  |
| **RB1** | 13q14.2 |  |  |  |  |
| **CDH1** | 16q22.1 |  |  |  |  |
| **EMP3** | 19q13.3 |  |  |  |  |
