# Supplementary material for: Delineating the Cytogenomic and Epigenomic Landscapes of Glioma Stem Cell Lines
Source: PLoS One. 2013 Feb 28;8(2):e57462. doi: 10.1371/journal.pone.0057462 (PMC3585345; doi:10.1371/journal.pone.0057462)
Supplement: Table S9 — A. Cancer de novo methylated genes: GSC and GBM FFPE tissues vs. foetal NSCs. B. De novo aberrantly methylated genes in GSC lines. (DOC) [file pone.0057462.s016.doc]

***Table S9. A.*** ***Cancer de novo methylated genes: GSC and GBM FFPE tissues vs. foetal NSCs.*** The significant GO annotations related to the nervous system, development and differentiation processes are listed. ***B. De novo aberrantly methylated genes in GSC lines.*** These genes are exclusively *de novo methylated* in GSC lines compared with foetal NSC lines.

| **A. Cancer *de novo* methylated genes: GSC and GBM FFPE tissues vs. foetal NSCs.** | | | |
| --- | --- | --- | --- |
| **Gene** | **Chromosomal location** | **Name** | **Significant GO terms related to the nervous system, development and differentiation** |
| *BARHL2** | 1p22.2 | BarH-like homeobox 2 | cell fate determination; [nervous system development](http://amigo.geneontology.org/cgi-bin/amigo/go.cgi?view=details&depth=1&query=7399); [neuron differentiation](http://amigo.geneontology.org/cgi-bin/amigo/go.cgi?view=details&depth=1&query=30182); [neuron migration](http://amigo.geneontology.org/cgi-bin/amigo/go.cgi?view=details&depth=1&query=1764) |
| *SIX2** | 2p21 | SIX homeobox 2 | [anatomical structure morphogenesis](http://amigo.geneontology.org/cgi-bin/amigo/go.cgi?view=details&depth=1&query=9653), [multicellular organism development](http://amigo.geneontology.org/cgi-bin/amigo/go.cgi?view=details&depth=1&query=7275) |
| *TBR1* | 2q24 | T-box, brain, 1 | [axon guidance](http://amigo.geneontology.org/cgi-bin/amigo/go.cgi?view=details&depth=1&query=7411); [brain development](http://amigo.geneontology.org/cgi-bin/amigo/go.cgi?view=details&depth=1&query=7420); [hindbrain development](http://amigo.geneontology.org/cgi-bin/amigo/go.cgi?view=details&depth=1&query=30902) |
| *GHSR* | 3q26.31 | growth hormone secretagogue receptor | none |
| *NKX3-2* | 4p16.3 | NK3 homeobox 2 | none |
| *PHOX2B** | 4p12 | paired-like homeobox 2b | [cell development](http://amigo.geneontology.org/cgi-bin/amigo/go.cgi?view=details&depth=1&query=48468); [cell differentiation in hindbrain](http://amigo.geneontology.org/cgi-bin/amigo/go.cgi?view=details&depth=1&query=21533); [glial cell differentiation](http://amigo.geneontology.org/cgi-bin/amigo/go.cgi?view=details&depth=1&query=10001); [multicellular organism development](http://amigo.geneontology.org/cgi-bin/amigo/go.cgi?view=details&depth=1&query=7275); [nervous system development](http://amigo.geneontology.org/cgi-bin/amigo/go.cgi?view=details&depth=1&query=7399); [neuron migration](http://amigo.geneontology.org/cgi-bin/amigo/go.cgi?view=details&depth=1&query=1764); [positive regulation of neuron differentiation](http://amigo.geneontology.org/cgi-bin/amigo/go.cgi?view=details&depth=1&query=45666) |
| *TEC* | 4p12 | tec protein tyrosine kinase | none |
| *PITX2** | 4q25 | paired-like homeodomain 2 | [multicellular organism development](http://amigo.geneontology.org/cgi-bin/amigo/go.cgi?view=details&depth=1&query=7275) |
| *MAB21L2* | 4q31 | mab-21-like 2 (C. elegans) | [multicellular organism development](http://amigo.geneontology.org/cgi-bin/amigo/go.cgi?view=details&depth=1&query=7275)  [nervous system development](http://amigo.geneontology.org/cgi-bin/amigo/go.cgi?view=details&depth=1&query=7399) |
| *NEUROG1** | 5q23-q31 | neurogenin 1 | [cell fate commitment](http://amigo.geneontology.org/cgi-bin/amigo/go.cgi?view=details&depth=1&query=45165); [multicellular organism development](http://amigo.geneontology.org/cgi-bin/amigo/go.cgi?view=details&depth=1&query=7275); [nervous system development](http://amigo.geneontology.org/cgi-bin/amigo/go.cgi?view=details&depth=1&query=7399); [neurogenesis](http://amigo.geneontology.org/cgi-bin/amigo/go.cgi?view=details&depth=1&query=22008); [positive regulation of neuron differentiation](http://amigo.geneontology.org/cgi-bin/amigo/go.cgi?view=details&depth=1&query=45666) |
| *ETV7** | 6p21 | ets variant 7 | none |
| *TBX18** | 6q14-q15 | T-box 18 | [multicellular organism development](http://amigo.geneontology.org/cgi-bin/amigo/go.cgi?view=details&depth=1&query=7275) |
| *HOXA9** | 7p15.2 | homeobox A9 | none |
| *TBX20** | 7p14.3 | T-box 20 | none |
| *MOGAT3* | 7q22.1 | monoacylglycerol O-acyltransferase 3 | none |
| *MIR183* | 7q32.2 | microRNA 183 | none |
| *PRDM14* | 8q13.3 | PR domain containing 14 | [cell fate specification](http://amigo.geneontology.org/cgi-bin/amigo/go.cgi?view=details&depth=1&query=1708); [cell morphogenesis](http://amigo.geneontology.org/cgi-bin/amigo/go.cgi?view=details&depth=1&query=902) |
| *DMRT2** | 9p24.3 | doublesex and mab-3 related transcription factor 2 | none |
| *DMRT3** | 9p24.3 | doublesex and mab-3 related transcription factor 3 | [cell differentiation](http://amigo.geneontology.org/cgi-bin/amigo/go.cgi?view=details&depth=1&query=30154); [multicellular organism development](http://amigo.geneontology.org/cgi-bin/amigo/go.cgi?view=details&depth=1&query=7275) |
| *FOXE1** | 9q22 | forkhead box E1 (thyroid transcription factor 2) | none |
| *HMX2** | 10q26.13 | H6 family homeobox 2 | none |
| *GSC** | 14q32.1 | goosecoid homeobox | multicellular organism development |
| *ALDH1A2* | 15q21.3 | aldehyde dehydrogenase 1 family, member A2 | none |
| *DSC3* | 18q12.1 | desmocollin 3 | none |
| *SALL3* | 18q23 | sal-like 3 (Drosophila) | none |
| *NKPD1* | 19q13.32 | NTPase, KAP family P-loop domain containing 1 | none |
| *ESX1* | Xq22.1 | ESX homeobox 1 | none |
| **B. *De novo* aberrantly methylated genes in GSC lines.** | | | |
| **Gene** | **Chromosomal location** | **Name** | **Significant GO terms related to the nervous system, development and differentiation** |
| *CACNA1E* | 1q25-q31 | calcium channel, voltage-dependent, R type, alpha 1E subunit | synaptic transmission |
| *ECEL1* | 2q37.1 | endothelin converting enzyme-like 1 | [neuropeptide signaling pathway](http://amigo.geneontology.org/cgi-bin/amigo/go.cgi?view=details&depth=1&query=7218) |
| *RPL26L1* | 5q35.1 | ribosomal protein L26-like 1 | none |
| *PTPRK* | 6q22.2-q22.3 | protein tyrosine phosphatase, receptor type, K | none |
| *TWIST1** | 7p21.2 | twist homolog 1 (Drosophila) | anatomical structural development; cell differentiation; multicellular organism development |
| *NEFL* | 8p21 | neurofilament, light polypeptide | [anterograde axon cargo transport](http://amigo.geneontology.org/cgi-bin/amigo/go.cgi?view=details&depth=1&query=8089); [axon transport of mitochondrion](http://amigo.geneontology.org/cgi-bin/amigo/go.cgi?view=details&depth=1&query=19896); [intermediate filament organization](http://amigo.geneontology.org/cgi-bin/amigo/go.cgi?view=details&depth=1&query=45109)  [neurofilament bundle assembly](http://amigo.geneontology.org/cgi-bin/amigo/go.cgi?view=details&depth=1&query=33693); [neurofilament bundle assembly](http://amigo.geneontology.org/cgi-bin/amigo/go.cgi?view=details&depth=1&query=33693); [retrograde axon cargo transport](http://amigo.geneontology.org/cgi-bin/amigo/go.cgi?view=details&depth=1&query=8090); [synaptic transmission](http://amigo.geneontology.org/cgi-bin/amigo/go.cgi?view=details&depth=1&query=7268) |
| *SYT10* | 12p11.1 | synaptotagmin X | none |
| *ISL2** | 15q23 | ISL LIM homeobox 2 | multicellular organism development |
| *STAC2* | 17q12 | SH3 and cysteine rich domain 2 | none |
| *SIM2** | 21q22.13 | single-minded homolog 2 (Drosophila) | [cell differentiation](http://amigo.geneontology.org/cgi-bin/amigo/go.cgi?view=details&depth=1&query=30154); [embryonic pattern specification](http://amigo.geneontology.org/cgi-bin/amigo/go.cgi?view=details&depth=1&query=9880); [multicellular organism development](http://amigo.geneontology.org/cgi-bin/amigo/go.cgi?view=details&depth=1&query=7275) |

*genes targeted by Suz12 in ES cells [1].

[1] Lee TI, Jenner RG, Boyer LA, Guenther MG, Levine SS, et al. (2006) Control of developmental regulators by Polycomb in human embryonic stem cells. Cell 125: 301-313.
