# Supplementary material for: Delineating the Cytogenomic and Epigenomic Landscapes of Glioma Stem Cell Lines
Source: PLoS One. 2013 Feb 28;8(2):e57462. doi: 10.1371/journal.pone.0057462 (PMC3585345; doi:10.1371/journal.pone.0057462)
Supplement: Table S10 — New ‘exclusive’ CNAs of GSC lines identified through a comparison with literature data. (DOC) [file pone.0057462.s017.doc]

***Table S10. New “exclusive” CNAs of GSC lines identified through a comparison with literature data.***

| **Cytoband** | **Position (Mb)** | | **GSC lines** | | | |
| --- | --- | --- | --- | --- | --- | --- |
|  | **from** | **to** | **Amplification** | **Gain** | **Deletion** | **Loss** |
| 1p36.21-p36.13 | 14,17 | 17,27 |  |  |  | GBM7 |
| 1p36.13 | 17,31 | 17,96 | GBM7 |  |  |  |
| 1p35.1-p34.3 | 32,35 | 38,36 |  |  |  | GBM7 |
| 1p35.1-p34.3 | 33,56 | 34,44 |  |  | GBM7 |  |
| 1p34.3 | 36,84 | 37,66 |  |  | GBM7 |  |
| 1p13.3 | 109,62 | 109,68 |  | GliNS2 |  |  |
| 1q42.13 | 228,32 | 228,44 |  |  |  | GBM7 |
| 1q44 | 246,54 | 247,18 |  | GliNS2 |  |  |
| 1q44 | 247,07 | 247,18 |  |  |  | GBM2 |
| 2p22.3 | 25,61 | 26,4 |  | GBM7 |  |  |
| 2p23.2 | 28,64 | 28,72 |  | GliNS2 |  |  |
| 2p22.3 | 32,04 | 32,81 |  | GBM7 |  |  |
| 2p13.1-p11.2 | 74,97 | 84,78 |  |  |  | GBM2 |
| 2p11.2 | 85,41 | 85,64 |  | GBM7 |  |  |
| 2p11.2 | 86,11 | 86,18 |  |  |  | GliNS2 |
| 2q11.2 | 96,8 | 96,91 |  |  |  | G179 |
| 2q12.1 | 102,16 | 102,8 |  |  |  | GBM2 |
| 2q14.2 | 119,96 | 120,15 | GBM2 |  |  |  |
| 2q22.1-q23.1 | 137,1 | 148,4 |  |  |  | GBM2 |
| 2q33.3-q35 | 208,98 | 216,44 |  |  |  | GliNS2 |
| 2q37.1 | 231,63 | 231,91 |  |  |  | G166 |
| 3p21.1 | 52,52 | 52,56 |  | GliNS2 |  |  |
| 3p21.1-p14.3 | 53,51 | 56,31 |  |  |  | GBM2 |
| 3p14.3 | 57,08 | 57,17 |  | GliNS2 |  |  |
| 3q25.1-q25.33 | 153,32 | 160,03 |  |  |  | G179 |
| 5p14.1-p13.3 | 26,98 | 29,94 |  |  |  | GliNS2 |
| 5p13.3 | 31,45 | 32,63 |  | GBM2 |  |  |
| 5q11.2 | 50,71 | 50,73 |  |  |  | GBM2 |
| 6p12.1-p11.2 | 55,22 | 57,3 |  | G166 |  |  |
| 6q16.1 | 97,17 | 97,48 |  | G166 |  |  |
| 6q21 | 107,21 | 108,88 |  | GBM2 |  |  |
| 6q21 | 112,49 | 112,68 |  | GliNS2 |  |  |
| 8q22.3 | 103,64 | 104,17 |  | GBM7 |  |  |
| 8q22.3-q23.2 | 104,27 | 111,73 |  |  |  | G166 |
| 8q24.23-q24.3 | 139,22 | 146,25 |  |  |  | GBM2 |
| 9q31.1 | 103,24 | 103,5 |  |  |  | GBM7 |
| 10q11.21 | 43,67 | 44,42 |  |  | GBM2 |  |
| 10q22.1 | 72,13 | 73,25 |  |  |  | GBM7 |
| 10q23.32-q23.33 | 93,69 | 94,38 |  | G166 |  |  |
| 11p11.2 | 47,28 | 47,42 |  | GBM2 |  |  |
| 11p11.2-q11 | 48,04 | 55,46 |  |  |  | GBM2 |
| 12q24.11-q24.13 | 108,8 | 109,7 |  | GBM7 |  |  |
| 14q13.2 | 34,11 | 35,08 |  | GBM7 |  |  |
| 14q32.31-q32.33 | 101,41 | 102,57 |  | GBM7 |  |  |
| 16p13.11 | 15,44 | 15,87 |  | G166 |  |  |
| 16q21-q23.1 | 67,21 | 68,04 |  | GBM7 |  |  |
| 17p13.3 | 0,2 | 1,29 |  | GBM7 |  |  |
| 17p13.1 | 7,4 | 7,45 |  | GliNS2 |  |  |
| 17p11.2 | 17,76 | 17,91 |  | GliNS2 |  |  |
| 20p13-p12.3 | 3,04 | 6,04 |  | GBM7 |  |  |
| 20q21.3 | 61,63 | 62,13 |  |  |  | G179 |
| 21q22.2-q22.3 | 41,37 | 42,1 |  |  |  | G179 |
| Xp22.33-p11.22 | 2,71 | 52,71 |  |  |  | G179 |
| Xp22.31 | 7,28 | 7,84 |  |  |  | GBM2 |
| Xp22.11 | 23,84 | 24,14 |  | GBM7 |  |  |
| Xp11.21-p11.1 | 56,28 | 56,61 | G179 |  |  |  |
