# Supplementary material for: Delineating the Cytogenomic and Epigenomic Landscapes of Glioma Stem Cell Lines
Source: PLoS One. 2013 Feb 28;8(2):e57462. doi: 10.1371/journal.pone.0057462 (PMC3585345; doi:10.1371/journal.pone.0057462)
Supplement: Table S11 — Top 10 pathways associated to new ‘exclusive’ CNA regions. (DOC) [file pone.0057462.s018.doc]

***Table S11. Top 10 pathways associated to new “exclusive” CNA regions.*** Each pathway is associated with a p-value (calculated by Ingenuity Pathway Analysis, IPA, software), which indicates the probability that such association could have occurred by chance.

| **Canonical pathway** | **Genes (↑gain; ↓loss)** | **p-value** |
| --- | --- | --- |
| **PPAR signaling** | RAS↑;HSP90AA1↑;IL18RAP↑;IL1A↑;IL1B↑;IL1F5↑;IL1F6↑;IL1F7↑; IL1F8↑;IL1F9↑;IL1F10↑;IL1R1↑;IL1RAPL1↓;IL1RL1↑;IL1RL2↑; IL1LRN↑;INS↑;MAP4K4↑;NFKBIA↑;NR1H3↑;SOS2↑;TRAF6↑ | 1.94e-05 |
| **IL-6 signaling** | ELK1↓;HRAS↑;HSPB7↓;IL18RAP↑;IL1A↑;IL1B↑;IL1F5↑;IL1F6↑;IL1F7↑;IL1F8↑;IL1F9↑;IL1F10↑;IL1R1↑;IL1RAPL1↓;IL1RL1↑;IL1RL2↑;IL1RN↑;MAP4K4↑;NFKBIA↑;RRAS2↑;SOS2↑;TNFAIP6↑;TRAF6↑ | 3.41e-05 |
| **Il-10 signaling** | ELK1↓;IL18RAP↑;IL1A↑;IL1B↑;IL1F5↑;IL1F6↑;IL1F7↑;IL1F8↑;IL1F9↑; IL1F10↑;IL1R1↑;IL1RAPL1↓;IL1RL1↑;IL1RL2↑;IL1RN↑;MAP4K4↑; NFKBIA↑;TRAF6↑ | 7.96e-05 |
| **EIF2 signaling** | EIF5↑;EIF1AX↓;EIF2C1↓;EIF2C2↓;EIF2C3↓;EIF2C4↓;EIF2S3↑;EIF3E↓; EIF3F↑;EIF3I↓;EIF4A1↑;EIF4G2↑;HRAS↑;INS↑;PIK3C2A↑;PPP1CC↑; RRAS2↑;SOS2↑ | 9.57e-04 |
| **NF-kB signaling** | BMPR2↑;CASP8↑;HADAC1↓;HRAS↑;IL1A↑;IL1B↑;IL1F5↑;IL1F6↑; IL1F7↑;IL1F8↑;IL1F9↑;IL1F10↑;IL1R1↑;IL1LRN↑;INS↑;LCK↓; MAP4K4↑;NFKBIA↑;PIK3C2↑;RRAS↑;SIGIRR↑;TAB3↓;TANK↑; TLR7↓;TRAF3↑;TRAF6↑;ZAP70↑ | 2.76e-03 |
| **p38 MAPK signaling** | ATF2↑;CREB1↑;ELK1↓;ESPB7↓;IL18RAP↑;IL1A↑;IL1B↑;IL1F5↑; IL1F6↑;IL1F7↑;IL1F8↑;IL1F9↑;IL1F10↑;IL1R1↑;IL1RAPL1↓;IL1RL1↑; IL1RL2↑;IL1RN↑;STAT1↑;TRAF6↑ | 3.56e-03 |
| **LXR/LXR activation** | IL18RAP↑;IL1A↑;IL1B↑;IL1F5↑;IL1F6↑;IL1F7↑;IL1F8↑;IL1F9↑;IL1F10↑;IL1R1↑;IL1RAPL1↓;IL1RL1↑;IL1RL2↑;IL1RN↑;NR1H3↑ | 7.07e-03 |
| **Integrin signaling** | ACTR3↑;ARF6↑;ARPC2↑;ARPC3↑;CRK↑;HRAS↑;ILK↑;ITGA4↑; ITGA6↑;ITGAV↑;ITGB6↑;LIMS1↑;MYL2↑;NCK2↑;PARVA↑;PIK3C2A↑;PPP1CC↑;PTK2↓;RALB↑;RAP2B↓;RHAOG↑;RND3↑;RRAS2↑;SOS2↑; TSPAN↑;TSPAN7↓;TTN↑;WAS↓;WIPF1↑ | 1.65e-02 |
| **Regulation of eIF4 and p70S6K signaling** | EIF1AX↓;EIF2C1↓;EIF2C2↓;EIF2C3↓;EIF2C4↓;EIF2S3↑;EIF3E↓;EIF3F↑;EIF3I↓;EIF4A1↑;EIF4G2↑;HRAS↑;ITGA4↑;PI3KC2A↑;PPP2R5C↑; RRAS2↑;SOS2↑ | 2.92e-02 |
| **Ephrin receptor signaling** | ACTR3↑;ANGPT1↓;ARPC2↑;ARPC3↑;ATF2↑;CFL2↑;CREB1↑;CRK↑; CXCL12↓;CXCR4↑;EPHA2↓;EPHA4↑;EPHA10↓;FIGF↓;GRIN3A↓; GRINA↓;HRAS↑;ITGA4↑;MAP4K4↑;NCK2↑;PTK2↓;RRAS2↑;SOS2↑; WAS↓;WIPF1↑ | 3.14e-02 |
