# Supplementary material for: Delineating the Cytogenomic and Epigenomic Landscapes of Glioma Stem Cell Lines
Source: PLoS One. 2013 Feb 28;8(2):e57462. doi: 10.1371/journal.pone.0057462 (PMC3585345; doi:10.1371/journal.pone.0057462)
Supplement: Table S12 — List of Top 10 pathways influenced by DNA methylation pattern in GSCs. (DOC) [file pone.0057462.s019.doc]

***Table S12. List of Top 10 pathways influenced by DNA methylation pattern in GSCs.*** Each pathway is associated with a p-value (calculated by Ingenuity Pathway Analysis, IPA, software), which indicates the probability that such association could have occurred by chance.

| **Canonical pathway** | **Genes (↑unmethylated; ↓methylated gene promoters)** | **p-value** |
| --- | --- | --- |
| **Molecular mechanism of cancer** | ABL1↑;ADCY3↓;ADCY6↑;ADCY8↑;AKT1↑;AKT2↑;AKT3↑;APH1A↑; ARHGEF2↑;ARHGEF3↑;ARHGEF4↓;ARHGEF10↓;ARHGEF11↑; ARHGEF12↑;ATR↑;BCL2L1↑;BMP4↑;BMP7↑;BMP8B↑;CASP6↑; CASP9↑;CCND3↑;CCNE2↑;DCD25A↑;CDC25C↑;CDK4↑;CDK6↑; CDKN2C↑;CDKN2D↑;CHEK1↑;CREBBP↓;CTNND1↓;CYSC↑;DAXX↑; DIABLO↑;E2F1↑;E2F2↑;E2F5↑;E2F6↑;ELK1↑;FOXO1↑;FZD4↑;FZD5↓; FZD9↑;GAB2↑;GNA12↑;GNA13↑;GNA14↓;GNAQ↑;GRB2↑ | 2.48e-08 |
| **Cyclins and cell cycle regulation** | ABL1↑;ATR↑;CCNA2↑;CCNB3↓;CCND3↑;CCNE2↑;CDC25A↑;CDC2↑; CDK4↑;CDK6↑;CDKN2C↑;CDKN2D↑;E2F1↑;E2F2↑;E2F5↑;E2F6↑; GSK3B↑;HDAC2↑;HDAC5↑;HDAC7↑;HDAC11↑;SKP1↑;PPP2CA↑; PPP2CB↑;PPP2R1B↑;PPP2R2B↑;PPP2R5E↑;RAF1↑;SIN3A↑;TGFB1↑; TGFB2↑;TGFB3↑ | 3.9e-06 |
| **Chronic myeloid leukemia signaling** | ABL1↑;AKT1↑;AKT2↑;AKT3↑;BCL2L1↑;CDK4↑;CDK6↑;CHUK↑; E2F1↑;E2F2↑;E2F5↑;E2F6↑;GAB2↑;GRB2↑;HDAC2↑;HDAC5↑; HDAC7↑;HDAC11↑;MAP2K2↑;MAPK1↑;EVI1↑;MYC↑;PIK3C2B↑; PIK3R3↑;RAF1↑;RBL1↑;RELA↑;RRAS↑;SIN3A↑;SOS1↑;SOS2↑; STAT5B↑;TGFB1↑;TGFB2↑;TGFB3↑ | 1.48e-05 |
| **Cell cycle: G1/S checkpoint regulation** | ABL1↑;ATR↑;CCND3↑;CCNE2↑;CDC25A↑;CDK4↑;CDK6↑;E2F1↑; E2F2↑;E2F5↑;E2F6↑;GSK3B↑;HDAC2↑;HDAC5↑;HDAC7↑;HDAC11↑; SKP1↑;MAX↑;MYC↑;RBL1↑;SIN3A↑;TGFB1↑;TGFB2↑;TGFB3↑ | 1.61e-05 |
| **Breast cancer regulation by stathmin1** | ADCY3↓;ADCY6↑;ADCY8↑;ARHGEF2↑;ARHGEF3↑;ARHGEF4↓; ARHGEF10↓;ARHGEF11↑;ARHGEF12↑;CALM2↑;CCNE2↑;CDC2↑; E2F1↑;E2F2↑;E2F5↑;E2F6↑;GNA13↑;GNAQ↑;GNB1↑;GNB4↑;GNB5↑; GNB1L↑;GNB2L1↑;GNG13↑;GRB2↑;ITPR2↑;MAP2K2↑;MAPK1↑; PAK1↑;PIK3C2B↑;PIK3R3↑;PPP1CC↑;PPP1R12A↑;PPP1R14B↑; PPP1R3C↑;PPP2CA↑;PPP2CB↑;PPP2R2B↑;PPP2R5E↑;PRKAG2↑; PRKCZ↑;PRKD1↑;PRKD3↑;RAF1↑;RB1CC1↑;ROCK1ROCK2↑; RRAS↑;SHC1↑ | 2.41e-05 |
| **PI3K/AKT signaling** | AKT1↑;AKT2↑;AKT3↑;BCL2L1↑;CDC37↑;CHUK↑;FOXO1↑;GAB2↑; GRB2↑;GSK3A↑;GSK3B↑;GYS1↑;HSP90AA1↑;ITGA↑;JAK1↑; MAP2K2↑;MAP3K5↑;MAPK1↑;PIK3R3↑;PPP2CA↑;PPP2CB↑; PPP2R1B↑;PPP2R2B↑;PPP2R5E↑;PRKCZ↑;PTGS2↑;RAF1↑;RELA↑; RHEB↑;RRAS↑;SHC1↑;SOS1↑;SOS2↑;TSC1↑;TYK2↑;YWHAE↑; YWHAG↑;YWHAQ↑;YWHAZ↑ | 1.02e-04 |
| **Pancreatic adenocarcinoma signaling** | ABL1↑;AKT1↑;AKT2↑;AKT3↑;BCL2L1↑;BRCA2↓;CASP9↑;CDK4↑; E2F1↑;E2F2↑;E2F5↑;E2F6↑;ELK1↑;GRB2↑;HBEGF↑;JAK1↑; MAP2K2↑;MAPK1↑;MAPK12↑;NOTCH1↑;PIK3C2B↑;PIK3R3↑; PTGS2↑;RAF1↑;RALGDS↑;RELA↑;SIN3A↑;SMAD2↑;STAT1↑;STAT3↑;TGFA↑;TGFB1↑;TGFB2↑;TGFB3↑;TYK2↑;VEGFC↓ | 1.44e-04 |
| **Regulation of eIF4 and p70S6K signaling** | AKT1↑;AKT2↑;AKT3↑;EIF1AX↑;EIF2A↑;EIF2S2↑;EIF2S3↑;EIF3D↑; EIF3E↑;EIF3H↑;EIF4A3↑;EIF4G2↑;EIF4G3↑;GRB2↑;IRS1↓;ITGA5↑; MAP2K2↑;MAPK1↑;MAPK11↑;MAPK12↑;PABPC1↑;PAIP1↑; PIK3C2B↑;PIK3R3↑;PPP2CA↑;PPP2CB↑;PPP2R1B↑;PPP2R2B↑; PPP2R5E↑; PRKCZ↑;RAF1↑;RPS6↑;RRAS↑;SHC1↑;SOS1↑;SOS2↑ | 1.44e-04 |
| **Role of BRCA1 in DNA damage response** | ATR1↑;BARD1↑;BRCA2↓;BRIP1↑;C17Orf70↑;CHEK1↑;E2F1↑;E2F2↑; E2F5↑;E2F6↑;FANCA↑;FANCC↑;FANCF↑;FANCG↑;FANCL↑;HLTF↑; NBN↑;RBBP8↑;RBL1↑;RFC2↑;RFC4↑;STAT1↑ | 3.25e-04 |

***Table S12. Cont’d***

| **Canonical pathway** | **Genes (↑unmethylated; ↓methylated gene promoters)** | **p-value** |
| --- | --- | --- |
| **Ephrin receptor signaling** | ABI1↑;ABL1↑;ADAM10↑;AKT1↑;AKT2↑;AKT3↑;ARPC3↑;ATF2↑; CREB5↑;CXCR4↑;EFNA5↑;EFNB2↑;EPHA2↑;EPHA4↑;EPHA5↑; EPHB4↑;GNA12↑;GNA13↑;GNA14↓;GNAQ↑;GNB1↑;GNB4↑;GNB5↑; GNB1L↑;GNB2L1↑;GNG13↑;GRB2↑;ITGA5↑;MAP2K2↑;MAP3K14↑; MAPK1↑;PAK1↑;PAK6↑;PAK7↑;PTK2↑;PTPN13↑;RAF1↑;RGS3↓; ROCK1↑;ROCK2↑;RRAS↑;SH2D3C↑;SHC1↑;SORBS1↑;SOS1↑;SOS2↑; STAT3↑;VEGFC↓;WAS↑;WASL↑ | 3.92e-04 |
